# ATPase-Dependent Control of the Mms21 SUMO Ligase during DNA Repair

**DOI:** 10.1371/journal.pbio.1002089

**Published:** 2015-03-12

**Authors:** Marcelino Bermúdez-López, Irene Pociño-Merino, Humberto Sánchez, Andrés Bueno, Clàudia Guasch, Seba Almedawar, Sergi Bru-Virgili, Eloi Garí, Claire Wyman, David Reverter, Neus Colomina, Jordi Torres-Rosell

**Affiliations:** 1 IRBLLEIDA, Dept. Ciències Mèdiques Bàsiques, Universitat de Lleida, Lleida, Spain; 2 Department of Genetics and Radiation Oncology, Erasmus Medical Center, Rotterdam, The Netherlands; 3 Institut de Biotecnologia i Biomedicina, Dept. de Bioquímica i Biologia Molecular, Universitat Autònoma de Barcelona, Bellaterra, Spain; Max Planck Institute of Biochemistry, GERMANY

## Abstract

Modification of proteins by SUMO is essential for the maintenance of genome integrity. During DNA replication, the Mms21-branch of the SUMO pathway counteracts recombination intermediates at damaged replication forks, thus facilitating sister chromatid disjunction. The Mms21 SUMO ligase docks to the arm region of the Smc5 protein in the Smc5/6 complex; together, they cooperate during recombinational DNA repair. Yet how the activity of the SUMO ligase is controlled remains unknown. Here we show that the SUMO ligase and the chromosome disjunction functions of Mms21 depend on its docking to an intact and active Smc5/6 complex, indicating that the Smc5/6-Mms21 complex operates as a large SUMO ligase in vivo. In spite of the physical distance separating the E3 and the nucleotide-binding domains in Smc5/6, Mms21-dependent sumoylation requires binding of ATP to Smc5, a step that is part of the ligase mechanism that assists Ubc9 function. The communication is enabled by the presence of a conserved disruption in the coiled coil domain of Smc5, pointing to potential conformational changes for SUMO ligase activation. In accordance, scanning force microscopy of the Smc5-Mms21 heterodimer shows that the molecule is physically remodeled in an ATP-dependent manner. Our results demonstrate that the ATP-binding activity of the Smc5/6 complex is coordinated with its SUMO ligase, through the coiled coil domain of Smc5 and the physical remodeling of the molecule, to promote sumoylation and chromosome disjunction during DNA repair.

## Abstract 2

Conserved conformational joints in the Mms21-Smc5/6 complex allow coordination between its ATPase and E3 SUMO ligase activities, integrating its structural and signaling roles during DNA repair to allow correct chromosome separation.

## Author Summary

The modification of target proteins by conjugation to SUMO—a small protein that acts as a regulatory tag—is essential for maintaining the integrity of genomes in most eukaryotic organisms. One critical step during the attachment of SUMO is the activation of the enzymes that catalyze this reaction—E1, E2, and the SUMO ligases. However, we currently do not fully understand how the different enzymes in the SUMO pathway are regulated. The SUMO ligase Mms21 is known to bind to Smc5/6, a large protein complex involved in the structural maintenance of chromosomes. Both Mms21 and Smc5/6 counteract the accumulation of recombination intermediates, which otherwise join replicated chromosomes, preventing their separation. Not surprisingly, the few known targets of the Mms21 ligase are mostly related to the repair of sister chromatids by recombination. Here, we show that the Mms21 SUMO ligase needs to bind to the Smc5/6 complex to promote chromosome separation. We used two Mms21-dependent SUMO conjugation targets—Smc5 and cohesin—to study the connection between the Mms21’s SUMO ligase activity and its binding partner, Smc5/6. Our results indicated that Mms21 activation is tightly coordinated with the intrinsic ATPase function of the Smc5/6 complex. However, the SUMO ligase and the ATPase lie in different domains of the Smc5/6-Mms21 complex that are normally distant from each other; we show that communication between these enzyme sites is enabled by the presence of conserved joints, which we suggest allow the necessary conformational changes required for SUMO ligase activation. This coordination of activities is extremely helpful for the cell, enabling it to integrate a structural role on chromatin during DNA repair with a signaling function, thereby promoting correct separation of the chromosomes.

## Introduction

During mitotic division, cells dedicate a large part of their efforts to accurately maintain and transmit genetic material to their offspring. The Structural Maintenance of Chromosomes (SMC) complexes play key structural roles in chromosome organization and dynamics and are crucial to maintain the integrity of the genome [1]. SMC proteins are rod-shaped molecules with a long coiled coil that separates a hinge or dimerization domain at one end and a nucleotide binding domain (NBD) at the other. Eukaryotes encode three different SMC complexes, known as cohesin, condensin, and Smc5/6. Heterotypic interactions between hinge domains lead to the formation of V-shaped molecules, which then bind to a variable number of non-SMC proteins [2]. The coiled coil domain of SMC proteins displays a remarkable flexibility, most probably due to the presence of conserved disruptions, which allow SMC complexes to adopt a wide variety of conformations [3–6]. Dimerization through the hinge and persistent connection of the NBD heads by a kleisin subunit generate large ring-like structures able to bind chromatin [7,8].

Smc6 was originally isolated in *Schizosaccharomyces pombe* as *rad18*, a gene involved in DNA repair [9]. The complex has been subsequently shown to be required during DNA double-stranded break repair and in response to perturbed replication forks, by either preventing the accumulation of recombination intermediates or promoting their removal, thus allowing chromosome segregation [10–19]. All subunits of the Smc5/6 complex are essential for viability in budding yeast. The complex is composed of the Smc5-Smc6 heterodimer, plus 6 Non-Smc Elements (named *NSE1* to *NSE6*), which collectively regulate its function [20]. Nse4 binds to the ATPase head region and has been proposed to be its kleisin subunit [21,22]. Nse4 also interacts with the Nse1 and Nse3 subunits, which together function as an heterodimeric ubiquitin ligase [23]. The Nse5 and Nse6 subunits are the least conserved proteins in the complex and, in budding yeast, bind to the hinge domains of the SMCs [21]. Finally, the Nse2 subunit, also known as Mms21, docks to the middle of the coiled coil region in the Smc5 molecule. The N-terminal part of the protein contains the Smc5-interacting domain and is essential for cell viability; in contrast, the C-terminus codes for a SUMO ligase SPL-RING domain and only becomes critical under conditions of genotoxic stress [24–27]. The SUMO protein can be covalently conjugated to lysine residues trough an enzymatic cascade [28], requiring activation by an E1 enzyme, transfer of SUMO to the E2 (Ubc9), and final E2-dependent direct conjugation to the target protein, or in collaboration with E3 SUMO ligase enzymes (Siz1, Siz2, and Mms21 in budding yeast).

The small number of SUMO ligases, relative to the large population of E2 and E3s in the ubiquitin system, raises the question of how sumoylation is regulated. Actually, sumoylation of Mms21-dependent targets is up-regulated by DNA damage through an unknown mechanism [25,29]. Sumoylation is essential for DNA damage repair, and mutations in E1, E2, or SUMO all lead to genotoxic sensitivity [30]. In contrast, Mms21 is the only E3 in yeast that renders cells sensitive to DNA damage when mutated [31], highlighting its central role in DNA repair. Different lines of evidence suggest that Mms21 promotes DNA repair from its location on the Smc5/6 complex. First, inactivation of the Mms21-branch of the SUMO pathway leads to a general decrease in genome integrity, and this phenotype is shared with mutants in other subunits of the Smc5/6 complex [20,32]. Second, differently to the related Siz/PIAS E3 ligases, Mms21 lacks a DNA-binding domain, which suggests that it must dock to other proteins to reach its chromatin-associated targets. And third, the *mms21-M5* allele, which is partially affected in its binding to Smc5, is also sensitive to various DNA-damaging agents [24]. Although these observations suggest that Mms21 needs to bind Smc5 to promote DNA repair, it is currently unknown if the Smc5/6 complex controls the activity of its associated SUMO ligase.

To investigate the relation between Mms21-dependent sumoylation, the association of the ligase with the Smc5/6 complex, and its role in maintaining the integrity of the genome, we have analyzed mutants in the Smc5/6 complex that block Mms21-dependent sumoylation. Here we report that Mms21 needs to bind an active Smc5/6 complex to reach its sumoylation targets and to promote sister chromatid disjunction. We also provide evidence demonstrating that Mms21-dependent sumoylation is controlled distally by the ATPase activity in the Smc5/6 complex, which is part of the E3 ligase mechanism that promotes sumoylation. Furthermore, we show that specific articulations in the Smc5 coiled coil structure allow communication between the ATPase heads and Mms21 in order to enable the activation of the SUMO ligase. Our findings suggest that the ATP-dependent chromosome structural role of the Smc5/6 complex and its SUMO-ligase activity are coordinated to ensure proper chromosome segregation.

## Results

### Docking of the Mms21 Ligase to the Smc5/6 Complex Is Required for Chromosome Disjunction and Sumoylation of Mms21-Dependent Targets

**Fig 1:**
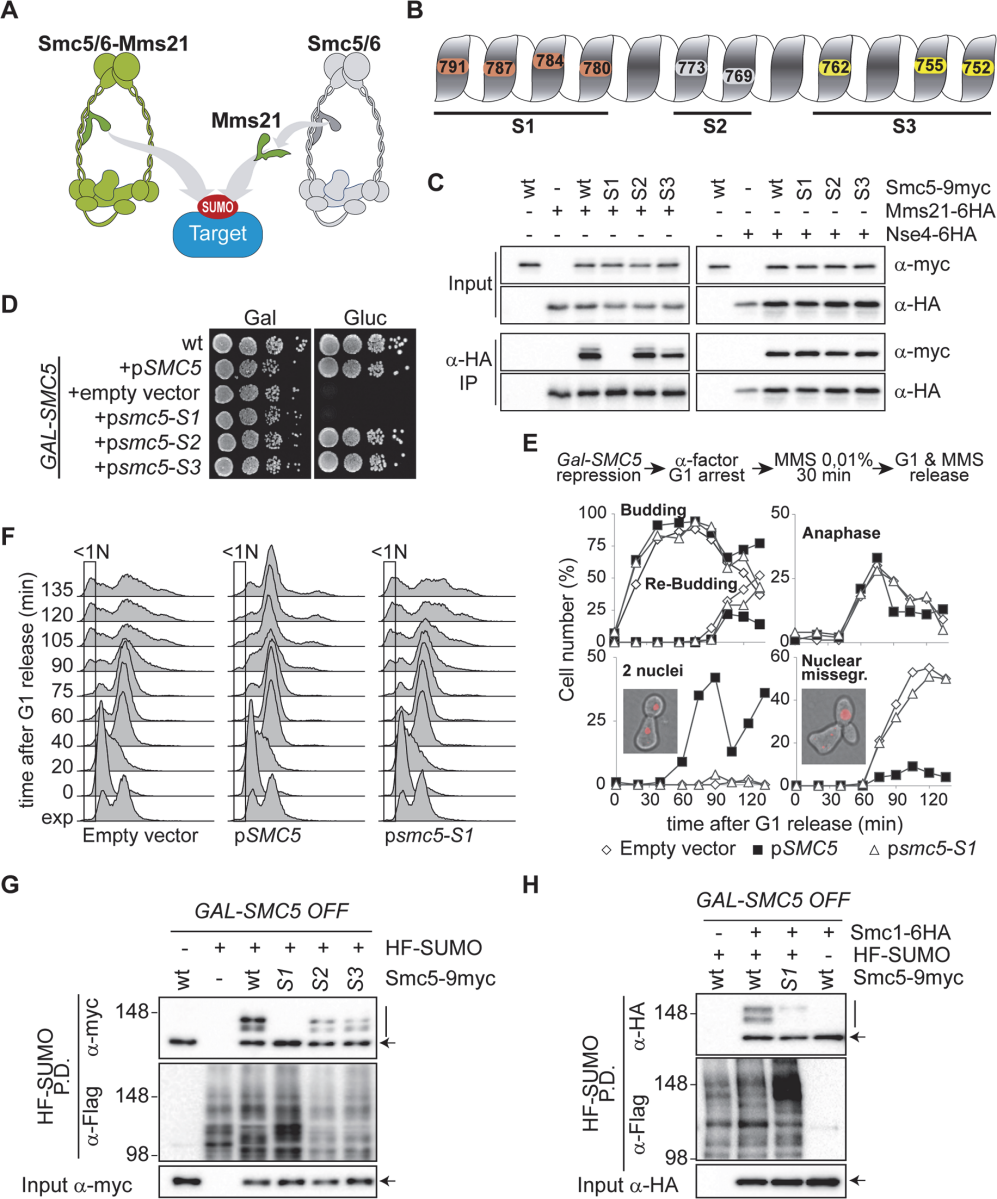
The Smc5-Mms21 interaction is required for sumoylation of Mms21 targets and chromosome segregation after DNA damage. **A**. Models for Mms21-dependent sumoylation: Mms21 may target proteins (including cohesin subunits) from its location in the Smc5/6 complex (left), or independently from Smc5/6 (right). **B**. Scheme of Mms21-binding surface on the coiled coil 2 of the Smc5 protein and location of mutated sites. **C**. Co-immunoprecipitation analysis of the Smc5-Mms21 and Smc5-Nse4 interactions. *MMS21-6HA* and *NSE4-6HA* cells were transformed with centromeric plasmids expressing the indicated *SMC5* alleles and subjected to anti-HA immunoprecipitation. **D**. Growth test analysis of *GALp-SMC5* cells transformed with the indicated centromeric plasmids. **E** and **F**. *GALp-SMC5* cells bearing the indicated vectors were shifted to glucose for 4 h to repress expression of the endogenous *SMC5* gene, and then treated as depicted in the figure; samples were taken at the indicated times for 4’,6-diamidino-2-phenylindole (DAPI) staining and microscopic examination (E) or Fluorescence-Activated Cell Sorting (FACS) analysis (F). Rectangles in F mark cells with less than 1N DNA content. **G**. *GALp-SMC5* cells ectopically expressing the indicated *SMC5* alleles from a centromeric vector were shifted to glucose for 6 h; 6xHis-Flag (HF) tagged SUMO was pulled down (P.D.) under denaturing conditions from yeast protein extracts (Input) to purify sumoylated species. Input and P.D. samples were analyzed by western blot with the indicated antibodies. **H**. *GALp-SMC5 SMC1-6HA* cells expressing the indicated *SMC5* alleles from a plasmid were shifted to glucose for 6 h. Protein extracts were processed for SUMO pull-down analysis as in G to analyze Smc1 sumoylation. In C, G, and H: wt = wild type, S1 = *smc5-S1*, S2 = *smc5-S2*, S3 = *smc5-S3*. In G and H, arrow points to unmodified form of the proteins, vertical bar to sumoylated forms; re-probing with anti-Flag is shown as a loading control for total SUMO in the purification.

The Mms21 SUMO ligase may promote sumoylation and chromosome disjunction from its location on the Smc5/6 complex, or independently from the complex (Fig. 1A). To test the relation between the docking state of Mms21 and its DNA repair and sumoylation functions, we generated mutants that disrupt the Smc5-Mms21 interaction. Since mutation of the SUMO ligase itself could potentially prevent its recruitment to other targets, we decided to disrupt the Smc5-Mms21 interaction by mutating the coiled coil domain of *SMC5*, without affecting the *MMS21* gene.

We designed three different sets of *smc5* mutants, namely *S1*, *S2*, and *S3*. Each of them carries mutations in residues lying on the surface of Smc5 that contacts Mms21 (Fig. 1B and S1 Fig.) [24]: *smc5-S1* has four mutations, I780R, I784R, F787A, and N791A; *smc5-S2* has two, M769A and K773A; and *smc5-S3* has three, Q752A, L755A, and L762A. The three *smc5* mutant alleles were fused to the 9myc epitope and expressed from centromeric vectors. Co-immunoprecipitation analysis confirmed that the smc5-S1 protein cannot interact with a 6HA-tagged wild-type Mms21 protein, while the *smc5-S3* mutation reduces the Smc5-Mms21 interaction to 50%, and the *smc5-S2* mutation does not seem to have any effect (Fig. 1C). In contrast, none of the *SMC5* mutants shows altered protein interactions with the Nse4 subunit of the Smc5/6 complex (Fig. 1C). To study their functionality, all mutants were ectopically expressed in *GALp-SMC5* cells, which allow conditional depletion of the endogenous *SMC5* gene. As shown in Fig. 1D, the *smc5-S2* and *smc5-S3* alleles can sustain growth of *GALp-SMC5* cells in glucose, while the *smc5-S1* mutant is lethal, further supporting the notion that the Smc5-Mms21 interaction is essential for cell viability [24].

SUMO-ligase impaired *mms21* mutant cells display chromosome segregation and disjunction defects after exposure to DNA damage [10], which are particularly severe in the ribosomal DNA (rDNA) array locus (S2 Fig.). To test if this is due to diminished sumoylation from the Smc5/6 complex, we arrested *GALp-SMC5* cells in G1 after switching off *SMC5* expression, and then treated them with 0.01% of MMS for 30 min before release into a synchronous cell cycle (Fig. 1E,F). All cultures entered the first and second cell cycles after the G1 arrest with similar kinetics, as evidenced by the appearance of budded and re-budded cells respectively. While cells ectopically expressing wild-type *SMC5* do not display any obvious mitotic defect, cells bearing an empty plasmid or expressing the *smc5-S1* allele show gross failures in chromosome segregation (Fig. 1E); this is evident as a slight increase in anaphase cells, the virtual absence of cells that have completed chromosome segregation (two nuclei), and the accumulation of cells with unequal separation of DNA masses between mother and daughter cells (nuclear missegregation). Additionally, FACS analysis shows the appearance of cells with less than 1N DNA content in cultures expressing no *SMC5* or the *smc5-S1* allele, indicative of chromosome segregation failures when Mms21 is not recruited to the Smc5/6 complex (Fig. 1F). The more severe effect of the *smc5-S1* mutation in chromosome segregation, relative to the SUMO-ligase defective *mms21Δc* mutant (S2B Fig.), is most probably due to the fact that the Smc5-Mms21 interaction is essential, while the SUMO ligase domain in Mms21 is not [24].

The experiments described above strongly support the idea that critical Mms21-dependent DNA repair targets are sumoylated from its location on the Smc5/6 complex, in contrast to a model where Mms21 sumoylates the repair targets independently from the rest of the complex (Fig. 1A). To directly test sumoylation of Mms21 targets, SUMO-conjugates were purified from cells that carry an N-terminal 6xHis-Flag (HF) epitope on the SUMO protein (Smt3). The endogenous wild-type Smc5 protein was depleted by shifting *GALp-SMC5* cells to glucose-containing media. The *smc5-S1* mutant protein displayed almost undetectable levels of sumoylation (Fig. 1G); on the other hand, sumoylation of Smc5 was slightly diminished, but not overly affected, by the *smc5-S2* or *smc5-S3* mutations, which do not substantially decrease its binding to Mms21. The sumoylation of the cohesin complex is also partially dependent on Mms21 [33,34], and this modification is required for recombination-dependent repair of chromosomes [15,34–36]. As shown in Fig. 1H, expression of the binding deficient *smc5-S1* allele severely impairs Smc1 sumoylation. In summary, these results confirm that Mms21 binding to the Smc5/6 complex is required for modification of known Mms21 substrates.

### Mms21-Mediated Sumoylation Requires an Active and Intact Smc5/6 Complex

**Fig 2:**
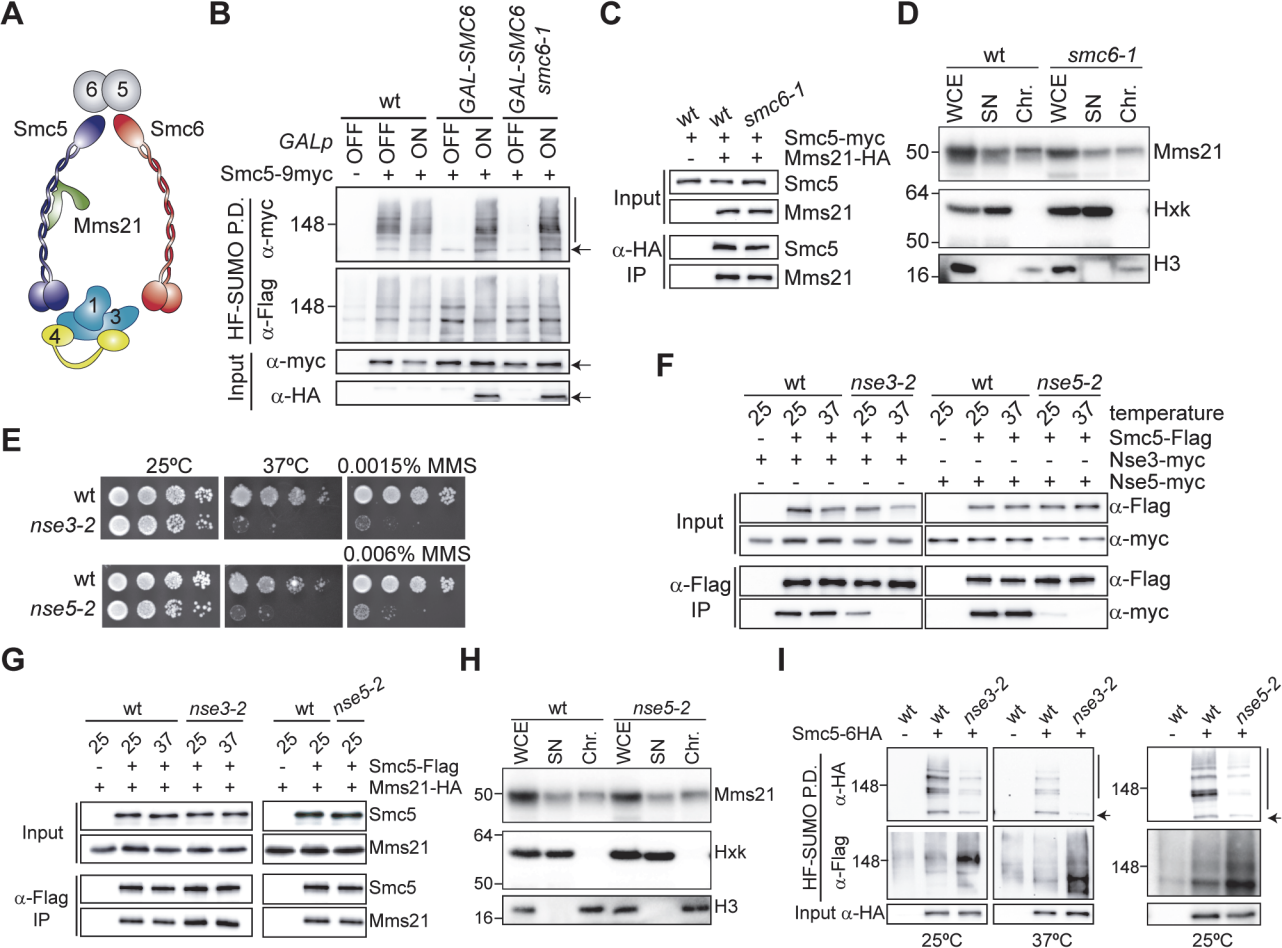
Mms21-dependent sumoylation requires an intact Smc5/6 complex. **A**. Composition of Smc5/6, depicting the different entities present in the complex. Nse subunits are labeled 1 to 6; Nse2 = Mms21. **B**. Sumoylation of Smc5 in *smc6* mutant cells. Samples of wild type and *GAL-3HA-SMC6* were collected from cells growing exponentially in galactose (*GALp* ON), or 12 h after shift to glucose to repress *3HA-SMC6* expression (*GALp* OFF). A *GALp-3HA-SMC6* strain expressing the *smc6-1* allele from a centromeric vector was also included in the analysis. Protein extracts were processed for HF-SUMO pull down as in Fig. 1G. **C**. Co-immunoprecipitation analysis of the Smc5-Mms21 interaction from wild type and *smc6-1* protein extracts. Wild type and *GALp-SMC6 smc6-1* cells expressing Smc5-9myc and Mms21-6HA were shifted to glucose for 12 h and processed for anti-HA immunoprecipitation. **D**. Chromatin fractionation assay from wild type and *smc6-1* cells to analyze the amount of chromatin-bound Mms21-6HA. Controls for a chromatin-bound protein (histone H3) and cytoplasmic soluble (Hexokinase; Hxk) proteins are shown. **E**. Temperature and methyl methanesulfonate (MMS)-sensitivity of *nse* hypomorphic alleles. Growth test of wild type, *nse3-2*, and *nse5-2* cells in YPD plates at 25°C (containing or not the indicated MMS concentration) or at 37°C. **F**. Analysis of the Smc5-Nse3 and Smc5-Nse5 interaction in *nse* hypomorphic alleles. Exponentially growing Smc5-6Flag cells, expressing 9myc-tagged versions of either the wild type or the indicated hypomorphic *nse* alleles, were shifted to 37°C for 2 h (37) or kept at 25°C (25) before Smc5-6Flag immunoprecipitation. **G**. Co-immunoprecipitation analysis of the Smc5-Mms21 interaction in *nse3-2* and *nse5-2* mutant cells. Smc5-6Flag was immunoprecipitated, as in F, from cells grown at the indicated temperatures. Co-immunoprecipitation of Mms21-6HA was analyzed by western blot. **H**. Chromatin fractionation assay from Mms21-6HA tagged wild type and *nse5-2* cells, as in D. **I**. HF-SUMO pull down from wild type, *nse3-2*, or *nse5-2* cells expressing Smc5-6HA, before and after a shift to 37°C. In B and G, arrow points to unmodified form of the proteins, vertical bar to sumoylated forms. In D and H, WCE: Whole Cell Extract; SN: Supernatant; Chr: Chromatin fraction.

The previous observations suggest that Mms21 binds the Smc5/6 complex to reach its substrates and promote DNA repair. Yet, and more appealingly, the structural and the SUMO-mediated signaling functions present in Smc5/6 might be coordinated to enhance DNA repair. The Smc5/6 complex can be dissected into different sub-entities (Fig. 2A) [24], including the Smc5-Smc6, Nse1-Nse3, and Nse5-Nse6 heterodimers, plus the Mms21 SUMO ligase and the Nse4 kleisin subunit. We therefore decided to test the participation of these sub-complexes on the E3 SUMO-ligase activity. Since Smc5 is a target of Mms21 [25] and the binding site for Mms21 in the complex [24], we used its sumoylation levels as an in vivo reporter for the activity of Mms21.

First, we tested cells that express their endogenous 3HA-tagged *SMC6* gene from the *GAL* promoter. We observed that turning off the *GAL* promoter leads to a drastic reduction in Smc5 sumoylation (Fig. 2B). Interestingly, Mms21 SUMO-ligase activity cannot be restored to wild-type levels by the hypomorphic *smc6-1* allele (Fig. 2B). Since the Mms21-Smc5 interaction is maintained, and Mms21 recruitment to chromatin is not overly affected in *smc6-1* cells (Fig. 2C and D), these results indicate that the Mms21 SUMO ligase must be inactive when *SMC6* function is impaired. Next, we studied the contribution of the Nse1-Nse3 and Nse5-Nse6 heterodimers using the thermosensitive and MMS-sensitive *nse3-2* and *nse5-2* hypomorphs (Fig. 2E). Co-immunoprecipitation analysis shows that the Smc5-Nse3 interaction is weaker in *nse3-2* cells at 25°C and becomes severely impaired upon shift to the restrictive temperature (Fig. 2F). The nse5-2 mutant protein also interacts weakly with Smc5, even at the permissive temperature (Fig. 2F). However, neither the *nse3-2* nor the *nse5-2* mutations affect the Smc5-Mms21 interaction (Fig. 2G), and the *nse5-2* mutation does not impair the binding of Mms21 to chromatin (Fig. 2H). Notably, both *nse3-2* and *nse5-2* mutant cells show reduced levels of Smc5 sumoylation, even at the permissive temperature for growth (Fig. 2I). These results indicate that proper recruitment of the Nse3 and Nse5 protein to Smc5/6 is required for the Mms21-dependent sumoylation of Smc5, in accordance with a previous report [37]. Finally, we confirmed that auxin-induced destruction of specific Smc5/6 subunits, including the Nse4 kleisin, also leads to a rapid loss of Smc5 sumoylation (S3 Fig.). Overall, our observations indicate that inactivation of different sub-entities in the Smc5/6 complex reduces Mms21-dependent sumoylation. Therefore, an active and intact Smc5/6 complex is required for the activity of its associated SUMO ligase.

### ATPase-Dependent Activation of the Mms21 SUMO-Ligase

**Fig 3:**
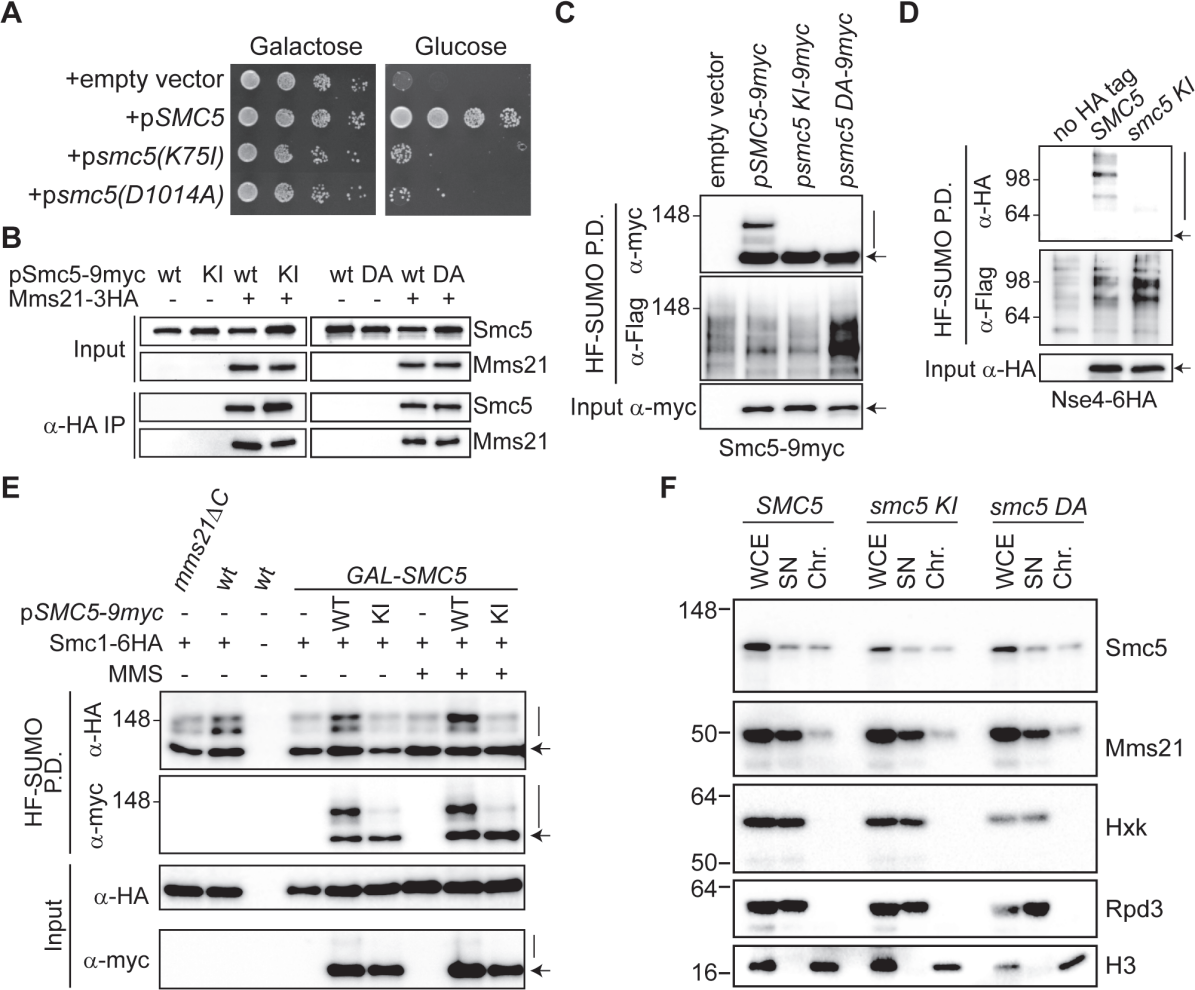
ATPase-dependent activity of the Mms21 SUMO ligase. **A**. Growth test of *GALp-SMC5* cells expressing wild-type *SMC5*, *smc5(K75I)*, or *smc5(D1014A)* from a centromeric vector in plates containing galactose (*GALp* ON) or glucose (*GALp* OFF). **B**. Mms21-3HA was immunoprecipitated from exponentially growing cells transformed with the indicated *SMC5*-expressing centromeric plasmids to test the Smc5-Mms21 interaction; wt = wild type; KI = *smc5(K75I)*; DA = *smc5(D1014A)*. **C**. Sumoylation analysis of ATPase-defective Smc5-9myc proteins. HF-SUMO pull-down analysis in wild-type cells transformed with plasmids expressing the indicated *SMC5* alleles. **D**. Sumoylation analysis of Nse4-6HA in *smc5* ATPase mutant cells. HF-SUMO pull-down analysis in *GALp-SMC5 NSE4-6HA* cells expressing the indicated *SMC5* alleles. Cells were shifted to glucose 6 h before collection to repress the endogenous *SMC5* gene. **E**. Sumoylation analysis of cohesin in *smc5* ATPase mutant cells. HF-SUMO pull down from cells of the indicated genotype (wt, *mms21ΔC* and *GALp-SMC5*), carrying a C-terminal 6HA tag on *SMC1*, and expressing or not an ectopic copy of *SMC5-9myc* (WT) or *smc5(K75I)-9myc* (KI) allele; where indicated, cells were treated with MMS 0,02% for 1 h (MMS) before collection. **F**. Chromatin fractionation assay from *GALp-SMC5 MMS21-6HA* cells expressing an ectopic 9myc-tagged copy of the indicated *SMC5* alleles, collected 6 h after shift to glucose to deplete the endogenous Smc5 protein. Controls for a chromatin-bound protein (histone H3), nuclear soluble (Rpd3) and cytoplasmic soluble (Hexokinase; Hxk) proteins are shown; WCE: Whole Cell Extract; SN: Supernatant; Chr: Chromatin fraction. In C–E, arrow points to unmodified Smc5, Nse4, or Smc1 proteins, and vertical bars to their sumoylated forms.

Since the essential function of SMC complexes in chromosome maintenance requires the ATPase activity of its SMC subunits, we introduced mutations in the Walker A or B ATPase motifs of Smc5 (K75I or D1014A, respectively) to compromise its binding to ATP. The ATPase-mutant alleles were expressed in *GALp-SMC5* cells and, as previously described, we observed that they render yeast cells non-viable (Fig. 3A) [38]. Co-immunoprecipitation experiments show that Mms21 binds with similar efficiency to either wild-type or ATPase mutant Smc5 proteins, indicating that there is no ATP-dependent modulation of the Smc5-Mms21 interaction (Fig. 3B). However, sumoylation of the ATPase-defective Smc5 proteins was almost undetectable (Fig. 3C), proving that they are not modified by their accompanying E3. As shown in Fig. 3D, the sumoylation deficiency affects other proteins in the complex, such as the Nse4 kleisin subunit, when cells only express the *smc5(K75I)* allele. Moreover, cells that only express the ATPase-defective version are also impaired in sumoylation of the Smc1 subunit of the cohesin complex (Fig. 3E), to a similar extent as *mms21* mutants lacking the C-terminal E3 ligase domain (*mms21ΔC*), indicating that the ATPase mutant complex is poised in an inactive state for sumoylation. Therefore, sumoylation of Smc5/6-Mms21 targets depends on the ATP-binding ability of Smc5. Although diminished sumoylation in the ATPase mutants could stem from defective recruitment of the complex to chromatin, this possibility seems unlikely, since in vitro experiments show that Smc5 binds efficiently to DNA in the absence of ATP [38], and we did not observe major alterations in the chromatin binding of the ATPase mutant proteins or the SUMO ligase using a chromatin fractionation assay (Fig. 3F), not even under conditions of competition with the endogenous wild-type Smc5 protein (S4 Fig.). These results further argue that the recruitment of Smc5/6 to chromatin does not depend on the sumoylation state of the complex.

### The ATPase Activity in the Smc5/6 Complex Is Part of the Ligase Mechanism that Triggers Sumoylation

**Fig 4:**
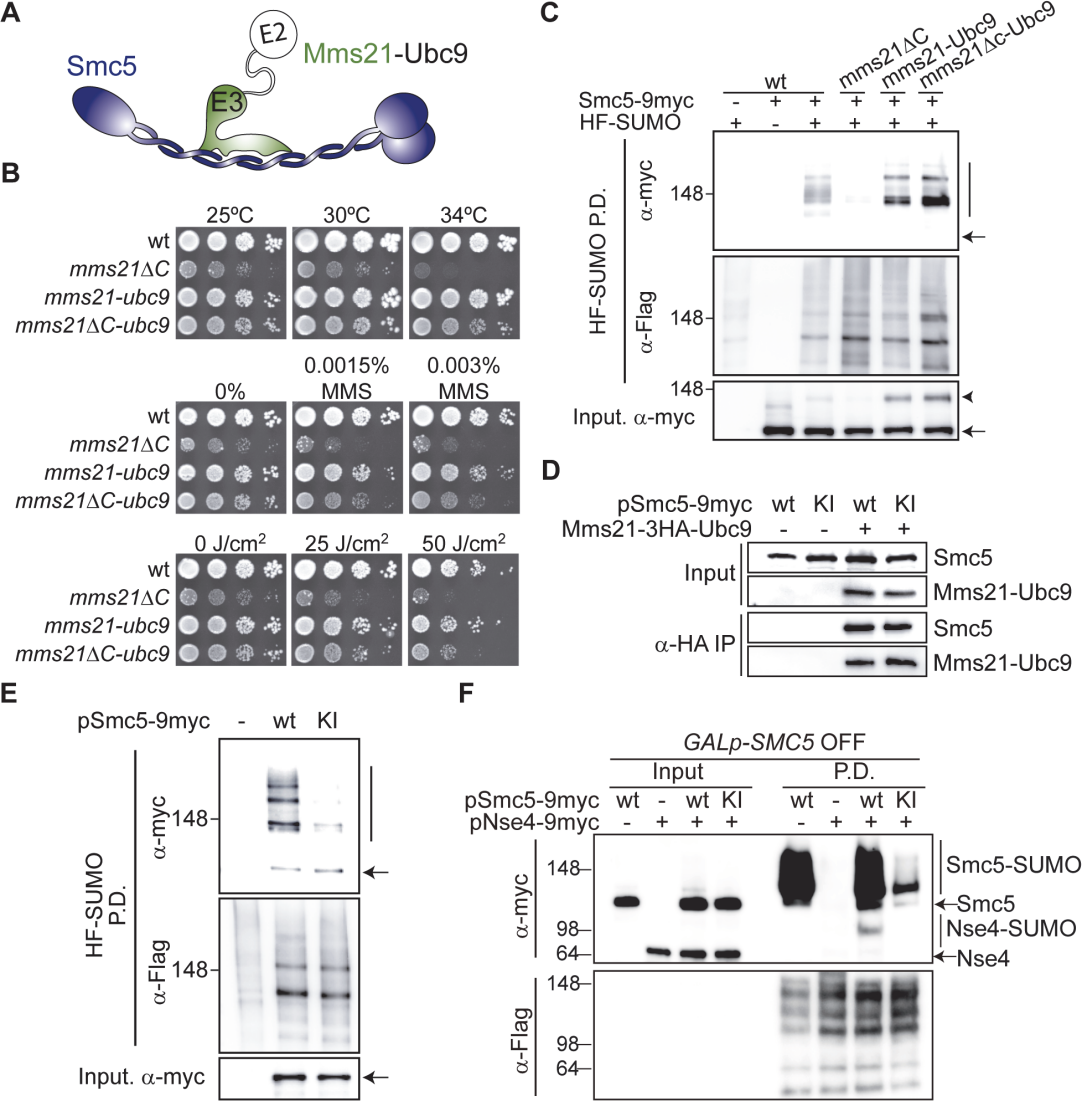
The ATPase in the Smc5/6 complex is part of the ligase mechanism that triggers sumoylation. **A.** Outline of an Mms21-Ubc9 (E3-E2) fusion, using a 3xHA linker, to force constitutive Ubc9 recruitment in the vicinity of the SUMO ligase. **B**. Growth test analysis of fusions of the E2 to a full-length Mms21 or to an *mms21Δc* allele lacking its C-terminal domain; plates were incubated at the indicated temperatures, at 25°C in the presence of MMS or at 25°C after irradiation with the indicated doses of ultraviolet (UV). **C**. Sumoylation analysis of Smc5-9myc under conditions of constitutive Ubc9 recruitment. HF-SUMO was pulled down from wt, *mms21ΔC*, *mms21-UBC9*, and *mms21Δc-UBC9* cells that also express a 9myc tagged version of its endogenous *SMC5* gene. **D**. Co-immunoprecipitation analysis of the E3-E2 binding to Smc5. Cells expressing or not an E3-E2 fusion from the *MMS21* locus and the indicated *SMC5* alleles from a centromeric plasmid, were grown to exponential phase and subjected to anti-HA immunoprecipitation to analyze binding of the fusion to Smc5-9myc. **E**. Sumoylation analysis of Smc5 under conditions of constitutive Ubc9 recruitment to the Smc5/6 complex. HF-SUMO was pulled down from E3-E2 cells expressing wild-type *SMC5-9myc* or ATPase-defective *smc5(K75I)-9myc* from a centromeric vector. **F**. Sumoylation analysis of Nse4 under conditions of constitutive Ubc9 recruitment to the Smc5/6 complex. HF-SUMO was pulled down from *GALp-SMC5 E3-E2* cells, expressing or not the indicated constructs from centromeric vectors, 6 h after shift to glucose to repress expression of the endogenous *SMC5* gene. Proteins were separated in a 4%–15% gradient gel. Note that sumoylation is much stronger for Smc5 than for Nse4. In C and E, arrow points to unmodified proteins; vertical bars are sumoylated forms. In C, arrowheads points to sumoylated Smc5 in the protein extract. In D–F, wt = wild type; KI = *smc5(K75I)*.

Mms21 could facilitate sumoylation-dependent DNA repair by bringing Ubc9 and its substrates into close proximity. In accordance, we observed that substitution of the SPL-RING sequence in *MMS21* for that of the E2 conjugating enzyme (*mms21ΔC-UBC9*) partially suppresses most of the *mms21ΔC* temperature and DNA damage sensitivities (Fig. 4A,B). As expected for constitutive Ubc9 recruitment to Smc5/6, substitution of the SPL-RING domain for Ubc9 not only restores, but actually up-regulates Smc5 sumoylation levels (Fig. 4C).

We speculated that the ATPase-inactive Smc5 mutant protein may fail to recruit Ubc9, thereby precluding sumoylation. If this hypothesis was correct, artificial recruitment of Ubc9 to Smc5/6 would eliminate the sumoylation differences between the wild-type and ATPase mutant Smc5 proteins. To explore this possibility, we integrated a second copy of *UBC9* fused to the C-terminus of the endogenous wild-type *MMS21* gene, using a 3xHA epitope as a linker (Fig. 4A). This fusion is functional and displays higher levels of Smc5 sumoylation, as expected from constitutive recruitment of both the E2 and E3 enzymes (Fig. 4B,C). Besides, the E3-E2 fusion co-immunoprecipitates similar amounts of both the ATPase active and inactive Smc5 proteins (Fig. 4D), indicating that the Smc5-Mms21 interaction is not affected in the ATPase mutant. Strikingly, and in spite of the constitutive binding of Ubc9 to the Smc5 protein, its full sumoylation still required an ATP-dependent step (Fig. 4E). To test the sumoylation of the non-SMC protein Nse4 in *E3-E2* cells, we integrated the Mms21-Ubc9 fusion in *GALp-SMC5* cells, and transformed them with centromeric vectors that express 9myc-tagged versions of the Nse4 and/or Smc5 proteins. As shown in Fig. 4F, we could not detect Nse4 sumoylation when no *SMC5* is expressed (second lane in Input and Pull Down); while ectopic expression of wild-type Smc5-9myc led to detectable levels of Nse4-9myc modification, the sumoylation of Nse4 could not be restored by expression of the ATPase-defective Smc5(K75I) allele. Altogether, these results suggest that the ATPase function of Smc5 is part of the ligase mechanism that enables sumoylation.

**Fig 5:**
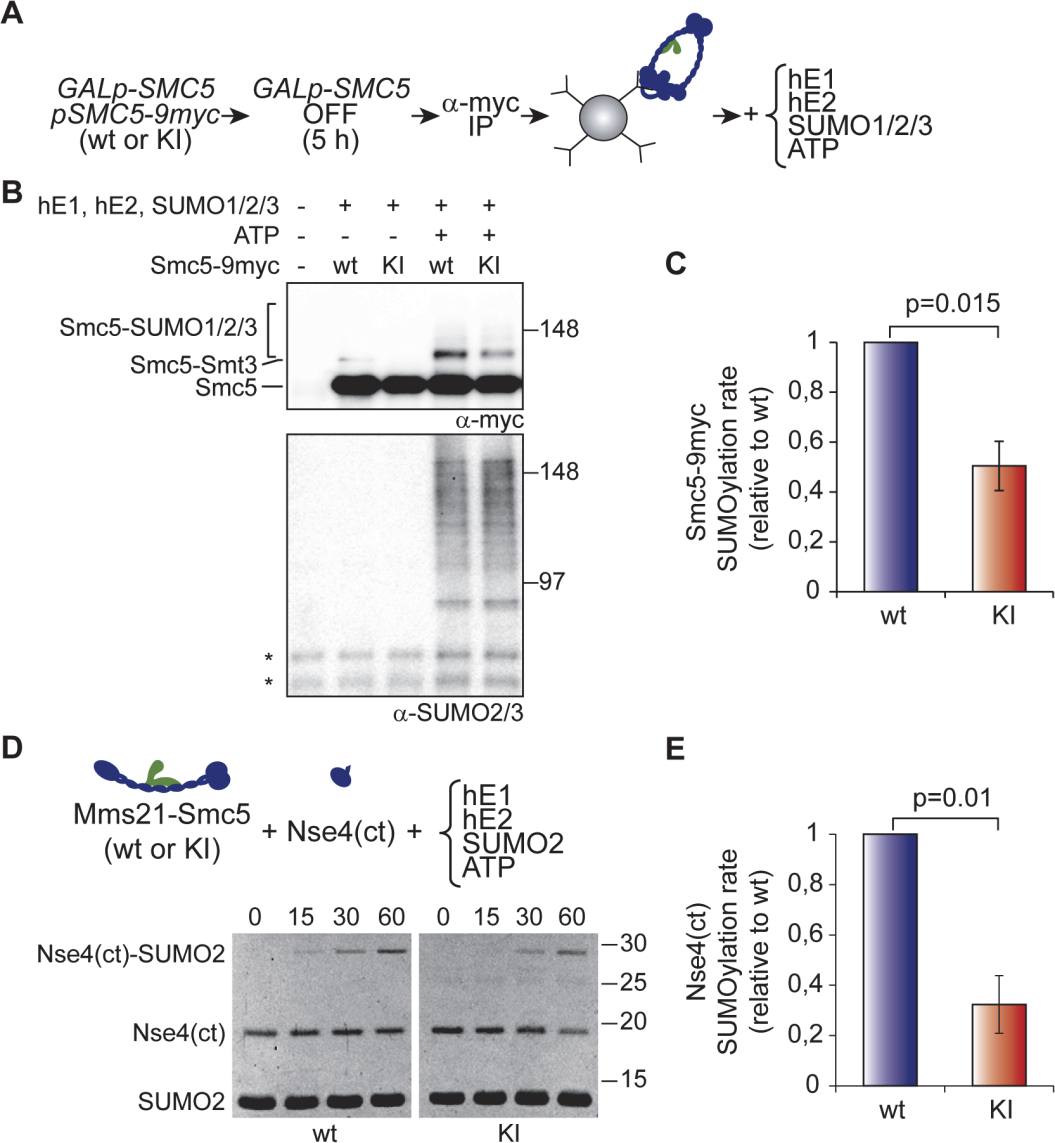
Binding of ATP to the ATPase head of Smc5 stimulates sumoylation in vitro. **A**. Experimental outline for the purification of wild-type or K75I mutant Smc5/6-Mms21 complexes used in the reactions. **B**. In vitro sumoylation reactions on immunoprecipitated Smc5-9myc. Reactions were stopped after 1 h of incubation at 37°C with the human E1, E2, and SUMO enzymes, as described in Materials and Methods, and analyzed by SDS-PAGE and immunoblotting using the indicated antibodies. **C**. Quantification of in vitro sumoylation rate in immunoprecipitated Smc5/6-Mms21 complexes, as described in Materials and Methods. Graph shows mean ± s.e.m.; *n* = 4; for each individual experiment, the rate of sumoylation for wild—type Smc5 was set to 1. **D**. In vitro sumoylation assay of the c-terminal domain (ct) of Nse4 (residues 246 to 402), using the Smc5-Mms21 heterodimer as the E3. Reactions were initiated by addition of ATP (time 0) and stopped at the indicated times. Samples were loaded in SDS-PAGE gels and stained with SYPRO-Ruby. **E**. Quantification of Nse4(ct) sumoylation rates, as described in Materials and Methods. Graph shows mean ± s.e.m.; *n* = 4; for each experiment, the rate of sumoylation using wild-type Smc5 was set to 1. wt = wild type; KI = *smc5(K75I)*. In B, asterisk marks unspecific band detected by the anti-SUMO2/3 antibody in immunoprecipitates.

To further analyze the participation of the ATPase heads on sumoylation, we immunoprecipitated active and inactive Smc5/6 complexes from yeast cells and tested their ability to sumoylate Smc5 in vitro (Fig. 5A). Addition of human E1, E2, and SUMO to the immunoprecipitates led to the appearance of sumoylated species and sumoylation of Smc5-9myc in an ATP-dependent manner (Fig. 5B). Interestingly, the ATPase mutant Smc5 protein can also be sumoylated in vitro, arguing that the Smc5(K75I) allele is not intrinsically unsumoylatable. However, we noticed that the rate of Smc5 sumoylation was significantly lower in the mutant than in the wild-type protein (Fig. 5B and C). A more severe effect was observed when the yeast E1, E2, and SUMO proteins were used in the assay (S5 Fig.). To focus on an even smaller number of components, we expressed and purified from *Escherichia coli* the C-terminal domain of the Nse4 kleisin (which interacts with the ATPase head of Smc5), and the Smc5-Mms21 heterodimer. We chose to co-express the heterodimer because monomeric Smc5 is poorly expressed in bacteria, while the Smc5-Mms21 pair accumulates at higher yields (S6 Fig.). Addition of ATP to the sumoylation reaction promoted the mono-sumoylation of the Nse4 fragment (Fig. 5D). Yet again, we detected a significant reduction in the rate of Nse4 sumoylation when the reaction proceeded in the presence of the mutant Smc5(K75I) protein, relative to wild-type Smc5 (Fig. 5E). Therefore the SUMO ligase is less active when the ATPase head cannot bind ATP. Overall, these results support the notion that, in vivo, the Smc5/6-Mms21 complex operates as a giant E3 SUMO ligase, which is sensitive to the ATPase activity of the SMC heads.

### The Smc5-Mms21 Heterodimer Undergoes ATP-Dependent Conformational Changes

**Fig 6:**
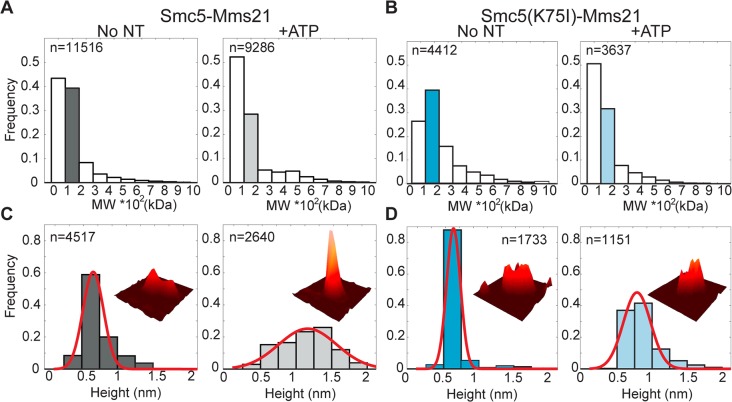
High-throughput SFM image analysis shows Smc5-Mms21 heterodimer is rearranged in an ATP-dependent manner. **A**. Volume distribution analysis of Smc5-Mms21 with (+ATP) or without ATP (No NT). **B**. Volume distribution analysis of Smc5(K75I)-Mms21 with (+ATP) or without ATP (No NT). **C**. Height distribution analysis of Smc5-Mms21 heterodimers between 100 and 200 kDa. Bins are highlighted with the same color as in panel A, dark and light gray, without and with ATP respectively. Average height of the 10% brighter pixels changed from 0.53 ± 0.2 nm (SD) without ATP to 1 ± 0.57 nm (SD) after ATP binding. **D**. Height distribution analysis of Smc5(K75I)-Nse2 heterodimers between 100 and 200 kDa (bins highlighted in the same color as in panel B, blue and light blue, without and with ATP respectively). Average height of the mutant heterodimer was 0.51 ± 0.14 nm (SD) without ATP and 0.66 ± 0.27 nm (SD) after ATP addition. n: number of analyzed particles; MW: molecular weight, kDa: kilo Daltons; nm: nanometers. Insets show representative SFM images (70x70 nm) of the heterodimers analyzed as 3-D view all at the same height (maximum height 1.5 nm). Red line is the normal distribution fit to data.

The SUMO ligase binds in the middle of the coiled coil domain of Smc5, at 16–24 nm from the ATPase heads [24], and yeast two-hybrid experiments indicate that Mms21 does not seem to contact the NBDs of the Smc5 protein [21]. Therefore, it is possible that the ATP-dependent communication between the NBDs and the SUMO ligase is triggered through conformational changes in the Smc5-Mms21 molecule. To test this hypothesis, the Smc5-Mms21 heterodimer was expressed in *E*. *coli*, purified, and imaged by scanning force microscopy (SFM) (Fig. 6 and S7 Fig.). Visual inspection of the purified particles shows the presence of approximately 50 nm rod-shaped structures, as expected for an SMC protein. In addition, we also observed many globular particles, suggesting that the coiled coil domain may fold about or wrap around the ATPase heads of Smc5, as occurs in other SMC proteins [39,40]. Particles were automatically recognized and classified according to volume. The volumetric distribution of wild type and ATPase K75I mutants shows that most particles have the expected dimensions for individual Smc5-Mms21 heterodimers. Prior incubation with ATP produces a shift towards smaller particles, which could reflect partial loss of the Smc5-Mms21 interaction, an effect that is observed in both the wild type and K75I mutant heterodimers (Fig. 6A and B). Interestingly, ATP increased the frequency particles with greater height, indicative of a more condensed shape and a conformational change (Fig. 6C). Monomeric Smc5 molecules are expected to have a low rate of ATP hydrolysis, suggesting that the observed conformational change takes place in response to ATP binding. As expected, mutation of the nucleotide binding domain in Smc5(K75I)-Mms21 heterodimers substantially reduced the degree of ATP-dependent compaction (Fig. 6D). The smaller conformational change observed in the K75I molecule might be due to partial binding of ATP on the Smc5(K75I) head. Therefore, binding to ATP induces a conformational change in the Smc5-Mms21 molecule.

### The Coiled Coil Domain of Smc5 Participates in the Activation of the Mms21 SUMO Ligase

Because it was not possible to directly visualize the coiled coil structure of Smc5 in most particles, we could not determine whether it participates in the ATP-dependent conformational change. On the other hand, if the coiled coil of Smc5 collaborates in the ATPase-dependent activation of its SUMO ligase, it should be possible to identify specific features in this domain that are critical for Mms21 activity.

**Fig 7:**
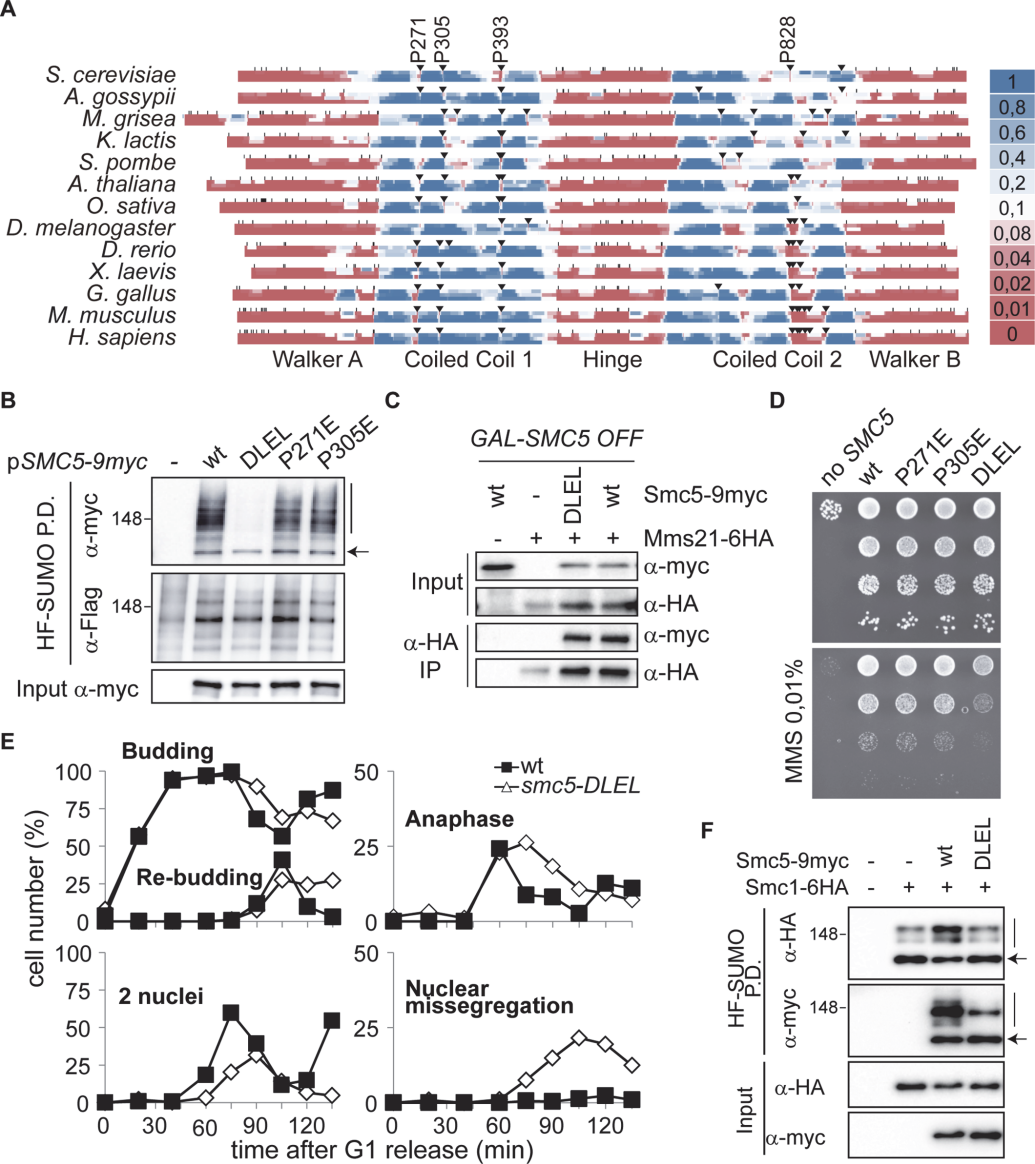
The coiled coil domain of Smc5 participates in activation of the Mms21 SUMO ligase. **A**. Coiled coil probability of the Smc5 protein sequence in different species (*Saccharomyces cerevisiae*, *Ashbya gossypii*, *Magnaporthe grisea*, *Kluyveromyces lactis*, *Schizosaccharomyces pombe*, *Arabidopsis thaliana*, *Oryza sativa*, *Drosophila melanogaster*, *Danio rerio*, *Xenopus laevis*, *Gallus gallus*, *Mus musculus*, *and Homo sapiens)*; sequences are aligned according to P393 position in budding yeast. Numerical values for coiled coil probability are colored as shown in the legend; small vertical lines mark position of proline residues, inverted arrowheads mark position of proline residues in coiled coils. **B**. HF-SUMO pull-down analysis from wild-type cells expressing the indicated *SMC5-9myc* alleles from a centromeric plasmid; *DLEL* mutant contains the H391D, P393E, and E394L mutations. **C**. Co-immunoprecipitation analysis of the Smc5-Mms21 interaction in wild-type and *smc5-DLEL* mutant cells. *GALp-SMC5* cells expressing wild-type *SMC5* or *smc5-DLEL* allele from a centromeric vector were shifted to glucose for 6 h before collection. Mms21-6HA was immunoprecipitated from protein extracts (input) with anti-HA beads (IP); samples were analyzed by SDS-PAGE and immunoblotting with the indicated antibodies. **D**. Growth test analysis of *GALp-SMC5* cells transformed with the indicated plasmids and plated in glucose-containing media at 30°C in the presence or absence of MMS 0.01%. **E**. Nuclear segregation defects in *smc5-DLEL* cells after DNA damage. Wild-type and *smc5-DLEL* cells were arrested in G1 with alpha factor, treated with MMS 0.01% for 30 min, and released into the cell cycle; samples were taken at the indicated times for microscopic analysis, as in Fig. 1E. **F**. HF-SUMO pull-down analysis in *GALp-SMC5 SMC1-6HA* cells expressing the indicated *SMC5-9myc* alleles from a centromeric vector; cells were shifted from galactose to glucose 6 h before collection to switch off the *GAL* promoter. In B and F, arrow points to unmodified SMC proteins; vertical bars are sumoylated forms.

Proline residues are rarely observed in coiled coils because they do not favor α-helical structures. Secondary structure prediction shows that, in most species, the probability of coiled coil drops at three different positions in the first coiled coil (CC1) of Smc5 (Fig. 7A). The first two disruptions in CC1 frequently involve a proline residue (P271 and P305). Mutation of these residues to glutamic acid, an abundant amino acid in the coiled coils of Smc5, had little effect on Smc5 sumoylation (Fig. 7B). The third disruption in CC1 contains a well-conserved proline residue (P393 in budding yeast; Fig. 7A). Different amino acids, besides P393, contribute to this disruption. We noticed that the combination of H391D, P393E, and E394L mutations, which locally change the coiled coil sequence from HLPE to DLEL (*smc5-DLEL*), restored the heptad periodicity and substantially increased the coiled coil probability (S8 Fig.). The *smc5-DLEL* mutation does not affect the interaction between Smc5 and Mms21 (Fig. 7C); in accordance, *smc5-DLEL* cells are viable, indicating that the essential function of Smc5 is not affected (Fig. 7D). However, *smc5-DLEL* cells are sensitive to MMS and exhibit nuclear segregation defects after a pulse of MMS in G1, suggesting improper disjunction of sister chromatids (Fig. 7D and E). Moreover, *smc5-DLEL* cells are compromised in Smc5 and cohesin sumoylation (Fig. 7B and F), indicating that mutation of the coiled coil disruption down-regulates the activity of the SUMO ligase.

**Fig 8:**
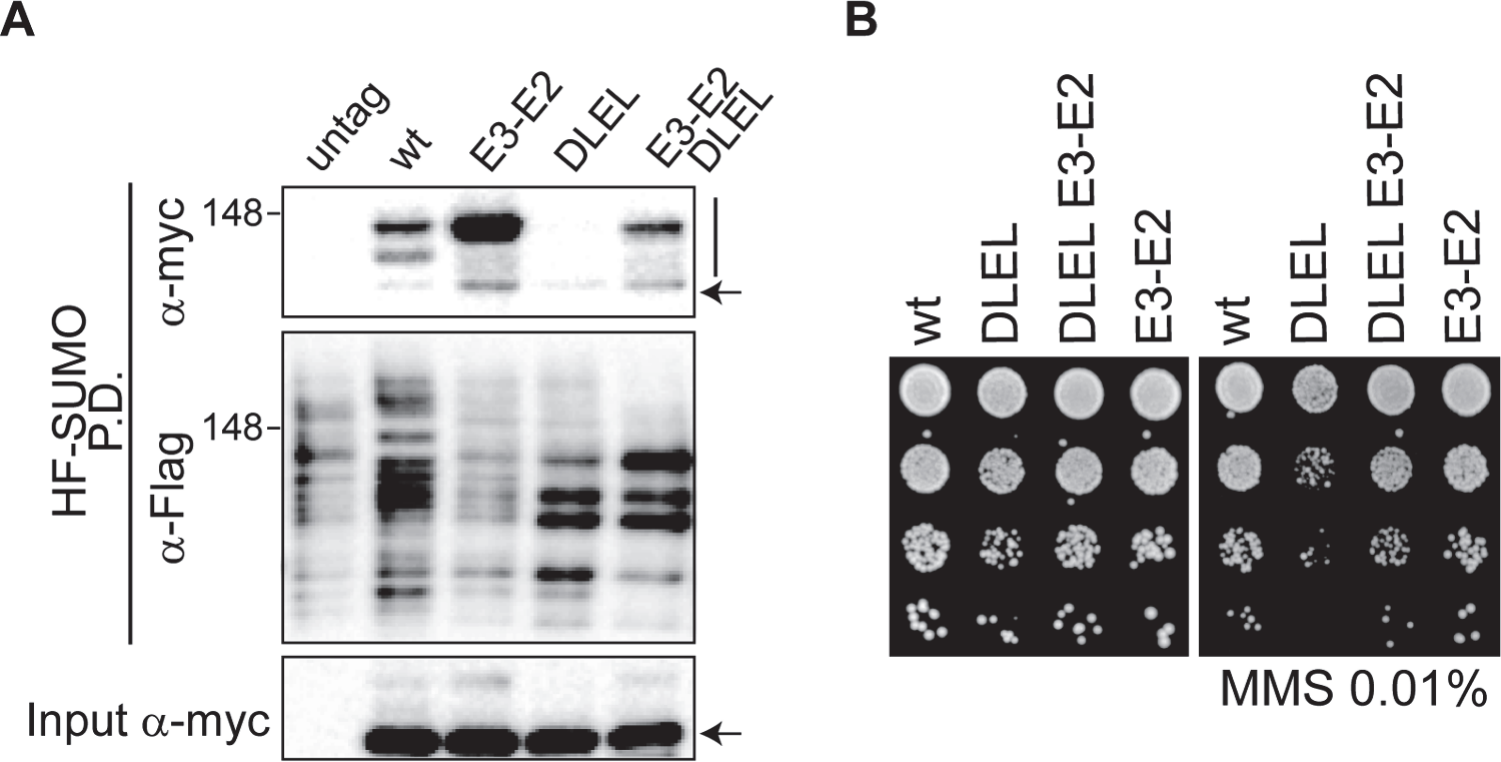
Up-regulation of Mms21-dependent sumoylation through expression of an E3-E2 fusion suppresses the smc5-DLEL coiled coil mutant. **A**. HF-SUMO pull-down analysis from wild-type or E3-E2 cells, expressing 9myc-tagged wild-type or *DLEL* mutant versions of the Smc5 protein form its endogenous location, as indicated. **B**. Growth test analysis of wild type, *E3-E2*, *smc5-DLEL*, and double *E3-E2 smc5-DLEL* mutant cells; plates were incubated at 30°C in the presence or absence of 0.01% MMS. In A, arrow points to unmodified Smc5; vertical bars are sumoylated forms.

To prove that the MMS-sensitivity of *smc5-DLEL* cells is directly related to impairment in SUMO-ligase activation and not to defective Smc5 function, we forced sumoylation by introducing the E3-E2 allele at the *MMS21* locus. We observed that constitutive recruitment of the E2 restores Smc5-DLEL sumoylation to levels comparable to wild-type cells (Fig. 8A). The fact that the double *smc5-DLEL E3-E2* mutant displays lower sumoylation than the single *E3-E2* is consistent with the idea that the third coiled coil disruption in Smc5 is also, as shown previously for the ATPase activity, part of the SUMO ligase activation mechanism. In accordance with restoration of Mms21-dependent sumoylation, constitutive E2 recruitment rescued the MMS sensitivity of *smc5-DLEL* cells (Fig. 8B), proving that their DNA damage sensitivity is not due to the structural alteration of the Smc5 protein, but to impaired activation of the SUMO ligase.

## Discussion

There is currently very little information about the regulation of SUMO enzymes. The Mms21 SUMO ligase is essential for the maintenance of genome stability [25–27,41], and it has been hypothesized that Mms21 docks to Smc5 to reach its few known substrates, which are mostly chromatin-associated [42]. Here we show that the SUMO ligase is physically and mechanistically coupled to the activity of Smc5/6. Remarkably, mutations that block Smc5 sumoylation also impinge on cohesin modification, which indicates that this mechanism is shared by other sumoylation targets outside the Smc5/6 complex.

In principle, cells could regulate Mms21-dependent sumoylation by triggering its recruitment on and off chromatin; for example, by binding to the Smc5/6 complex. However, our results indicate that the mere proximity of Mms21 is not sufficient for sumoylation of some of its targets. The clearest example is the Smc5 protein, which binds strongly to and is sumoylated by Mms21 [24,25]: ATPase-defective Smc5 proteins are not sumoylated, despite normal recruitment of both Smc5 and Mms21 to chromatin and proper binding of Smc5 to the SUMO ligase. Therefore, Smc5/6-Mms21 dependent sumoylation is only possible from an active Smc5 protein. Our findings do not exclude the possibility that Mms21 might target other proteins in an Smc5/6- (and hence ATPase-) independent manner. However, such targets do not seem to participate in chromosome disjunction, as a wild-type Mms21 protein is incapable of promoting chromosome segregation when not recruited to Smc5 (Fig. 1E and F). The *smc5-S1* mutant developed in this study should thus become an indispensable tool to test putative Smc5/6-independent roles of the Mms21 SUMO ligase.

Our results also shed new light on how the Smc5/6-Mms21 branch of the SUMO pathway is controlled at the molecular level. Differently to the ubiquitin pathway, Ubc9 can directly transfer SUMO to its targets [28]. However, sumoylation of most proteins in budding yeast requires the presence of a SUMO ligase [43]. In the case of substrates that directly interact with the E2, the presence of an E3 might promote the correct orientation of the Ubc9-SUMO thioester for catalysis [44] or establish additional contacts with the substrate [45]. Sumoylation of Smc5/6 subunits also requires binding of the E3 to its substrate (Fig. 1), suggesting that Mms21 promotes sumoylation and chromosome repair by stimulating the formation of an E2-SUMO-E3-target complex. In accordance, artificial recruitment of the E2 to Smc5/6 in cells lacking the E3 SUMO ligase domain of Mms21 suppressed their DNA-damage sensitivity (Fig. 4). Importantly, our results also point to an unforeseen regulation of Mms21 in the Smc5/6 complex, since the ATPase activity of Smc5 is required for activation of the Mms21 ligase, even after recruitment of the E2 (Figs. 3 and 4). The ATP-dependency is not restricted to the Smc5 protein, as Nse4 and cohesin (Fig. 3D and E) are also hypo-sumoylated when Smc5 cannot bind ATP. Therefore, the Smc5/6-Mms21 complex must have lower activity when Smc5 is not bound to ATP. In accordance, our in vitro assays show a 2- to 3-fold lower rate of sumoylation in ATPase-defective Smc5 mutant proteins. Such defect might seriously compromise the ability of Smc5 ATPase mutants to sustain proper sumoylation levels in vivo, as SUMO enzymes are probably less accessible than in vitro, and SUMO peptidases are actively removing SUMO from targets. Different situations could account for the lower sumoylation in ATPase mutants, ranging from the incapacity to properly orient the Ubc9-SUMO thioester, the inhibition of SUMO discharge from Ubc9, or the presence of a molecular obstruction in those Smc5/6 molecules that are not charged with ATP. A detailed view of the Mms21-Ubc9-SUMO interaction structure, as well as the participation of Smc5/6 structural elements in this process, will be required to solve this issue.

Apart from the ATPase heads, the coiled coil domain to which Mms21 binds is also required for SUMO ligase activation and chromosome segregation. To our knowledge, this is the first report for a specific function of a coiled coil domain in an SMC protein. Our results are consistent with the *smc5-DLEL* mutation severing the communication between the ATPase heads and Mms21. Although we cannot formally discard that the *DLEL* mutation indirectly decreases the ATPase activity, this situation seems unlikely because (i) the mutation is located far away from the NBDs of the Smc5 protein, and close to the Mms21 docking site; (ii) differently than ATPase mutants, *smc5-DLEL* cells are viable; and (iii) the MMS-sensitivity of the *DLEL* mutant can be bypassed by constitutive tethering of Ubc9 to the complex, an observation that directly links this coiled coil disruption to activation of the SUMO-ligase. Moreover, the inability of the *smc5-DLEL E3-E2* double mutant to reach the hyper-sumoylation state of the *E3-E2* single mutant shows that this knuckle is part of the E3. It is worth noting that the proline residue present in this disruption is conserved in evolution, indicative of a vital function and a possible similar regulation of the Smc5/6-Mms21 ligase in humans.

**Fig 9:**
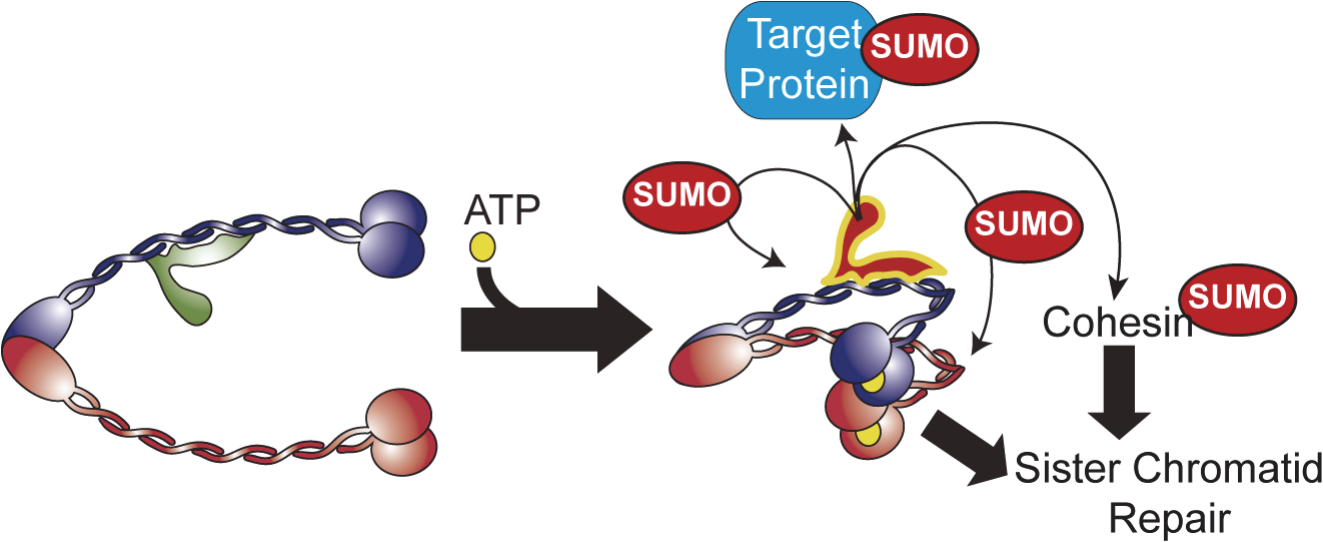
Model for the ATPase-dependent regulation of the SUMO ligase activity in the Smc5/6-Mms21 complex. Binding of ATP to the ATPase heads of Smc5/6 induces a conformational change that activates the Mms21 SUMO ligase; the structural maintenance of chromosomes (SMC) role of the Smc5/6 complex and the Mms21-dependent sumoylation of targets, such as cohesin, collaborate in homologous recombinational repair and chromosome disjunction.

The coiled coil domains in SMC proteins display a wide variety of conformations, most probably due to the presence of kinks at specific disruptions in this domain [39,40]. The coiled coil flexibility in SMC proteins might be important to accommodate chromatin fibers inside the ring structure, and it might also help to bring different domains of the molecule in close contact [46,47]. We have observed an analogous conformational heterogeneity for the Smc5-Mms21 heterodimer and further conformational changes upon ATP binding. ATPases are known to couple ATP binding and hydrolysis to mechanical work, and coiled coil domains can transmit this information to other regions of the molecule [48,49]. In the case of dynein, the communication is enabled by a change in the registry of the two short alpha-helical chains in the coiled coils [48], although this seems dubious for Mms21, given the distance separating the NBDs and the Mms21 binding site. Other possibilities could be the rotation of the ATPase heads along the coiled coils axis, as is the case for Rad50 [50] or folding of the molecule at specific articulated disruptions, as has been hypothesized for the cohesin complex [51]. The participation of the P393 disruption in Mms21-dependent sumoylation invokes a model where the ATP-dependent reshaping of the molecule allows activation of the SUMO ligase (Fig. 9).

If bending of the Smc5/6 molecule leads to Mms21 activation, this might only happen in the context of a competent Smc5-Smc6-Nse4 ring. Indeed, our results suggest that the Smc5/6 functions as a giant SUMO E3 enzyme, and the different sub-entities present in the Smc5/6 molecule are required for Mms21-dependent sumoylation. We hypothesize that the non-SMC elements, as well as Smc6, could directly participate in Mms21 activation, as we have shown here for Smc5 (Fig. 3 and 7). A second possibility is that the *NSE* mutants analyzed might diminish the ATPase activity of the complex. For example, the kleisin subunit in the cohesin complex is known to regulate the ATPase activity of the SMC heads [52], and loss of Nse4 could inactivate Smc5/6 in an analogous manner. A third option is that *NSE* malfunction might indirectly diminish the ATPase activity by precluding Smc5/6 recruitment to damaged DNA [37]. It has been proposed that the Nse5-Nse6 sub-complex might regulate chromatin association of Smc5/6 through opening of the Smc5-Smc6 hinge interface [21], analogously to what occurs during chromatin loading of cohesin [51]. Still, it is worth noting that Mms21 remains bound to chromatin in *nse5-2* mutants, and docked onto Smc5 (despite down-regulation of Smc5 sumoylation) in all the thermosensitive *smc5/6* mutants tested; the most plausible explanation being that Mms21 is not active. Interestingly, Nse5 is known to directly interact with proteins of the SUMO pathway, including Ubc9 and SUMO [37,53]; therefore, it is tempting to speculate that this sub-complex might play specific roles in SUMO conjugation through recruitment of Ubc9.

Our study emphasizes the importance of the intimate relation between the Mms21 SUMO ligase and its binding site, the Smc5/6 complex. The Mms21 branch of the SUMO pathway and the Smc5/6 complex are required to prevent the accumulation and/or promote the removal of pathological recombinogenic structures [10–13,16]. These structures are lethal, since they prevent segregation of sister chromatids. The meiotic program, which requires the induction of double-stranded breaks, also requires the Smc5/6-Mms21 complex to properly channel recombination intermediates [17–19]. Here we have shown that mis-regulation of the SUMO ligase activity in the complex renders cells unable to disjoin and segregate chromosomes after DNA damage. The integration of the ATPase and the SUMO ligase in the Smc5/6-Mms21 complex should help to coordinate a structural activity on chromosomes with a signaling role via sumoylation, both of which would be directed to the efficient resolution and proper segregation of sister chromatids. The relatively small number of known Mms21 targets, most of which participate in processing of double-stranded breaks, points in this direction. The case of cohesin is paradigmatic, as its Mms21-dependent sumoylation is known to be required for establishment of sister-chromatid cohesion and sister chromatid recombination [34–36]. The growing list of damage-induced targets of the Mms21 ligase should definitely contribute to our understanding of this branch of the SUMO pathway.

## Materials and Methods

### Yeast Growth Conditions

Yeast cells were grown in YP (Yeast extract Peptone), or minimum complete medium (SC) to select for plasmid auxotrophies, plus the indicated carbon source at 2% final concentration. For auxin-induced degrons, IAA (SIGMA) was added to 1 mM from a 0.5 M stock in water.

### Cell Cycle Experiments

Exponentially growing cells were arrested in G1 by addition of 10^–8^ M alpha factor (Genscript) at 30°C for 2 h or until >95% of cells were arrested in G1. Cells were then treated with 0.01% MMS (SIGMA) for 30 min to induce a pulse of alkylation damage, and cultures were released by washing cells three times and re-suspension in media containing 0.1 mg/ml pronase E (SIGMA). Synchronic cultures were routinely checked by FACS analysis. DNA was stained using 4,6,-Diamidino-2-phenylindole (DAPI) at 1 μg/ml final concentration in the presence of mounting solution and 0.4% Triton X-100 to permeablize cells. For fluorescence microscopy, series of z-focal plane images were collected with a DP30 monochrome camera mounted on an upright BX51 Olympus fluorescence microscope.

### Construction of Strains and Plasmids

Epitope tagging of genes and deletions were performed as described [54,55]. Fusion of genes to an auxin-induced degron was done as described [56]. *SMC5-9myc* was amplified by PCR from the yeast strain YTR914 (*SMC5-9myc*:*hphNT1*) and cloned into the SphI/KpnI sites in YCplac22. Then, the *ADH1p* promoter was cloned upstream of the *SMC5* gene by recombination cloning in *recA+* MC1061 cells to yield plasmid pTR1094 (YCplac22-*ADH1p-SMC5-9myc*). *MMS21* was cloned at the KpnI site in pTR797 (pYES2-3HA) and then moved to the SalI site in pRS315 to yield pNC2275 (pRS315-*GALp-MMS21-3HA*). All other *SMC5*- or *MMS21*-expressing plasmids used in this study are derived by site-directed mutagenesis from pTR1094 or pNC2275, respectively, using QuikChange XL (Stratagene). The E3-E2 strain was created by fusion PCR of two partially overlapping sequences, one containing an *MMS21-3HA-UBC9* sequence from plasmid pTR1138 (pYES2-*MMS21-3HA-UBC9*), the other an *hphNT* or *natNT* cassette for integration at the 3′ end of the *MMS21* gene. Transformants were checked by PCR and western blot. Oligos used for PCR are provided upon request.

### Immunoprecipitation and Western Blotting

Pull-down analysis of sumoylated proteins was performed essentially as described [34]. In all pull downs (except those shown in Figs. 2B, 2I, 4C, 4E, and **6B**), cells were denatured during harvesting, and prior to snap-freezing, by sequential resuspension of the yeast pellet in 12% TCA and in 1M Tris-HCl pH 8. Cells were mechanically broken in 8M urea, and incubated with Ni-NTA beads in the presence of 15mM imidazole overnight at room temperature. Bound proteins were eluted with SDS-PAGE loading buffer. In all cases, SUMO pull downs were loaded in SDS-PAGE gels next to protein extracts to confirm the slower mobility of SUMO conjugates with respect to the unmodified protein. All proteins were resolved in 10% SDS-PAGE gels, except SMC proteins (7.5%), histone H3 (15%), and Fig. 4F (4%–15% gradient gel; BioRad). For co-immunoprecipitation analysis, protein extracts were prepared in EBX as previously described [34]. myc-tagged proteins were immunoprecipitated using anti-myc antibodies (9E10, Roche) coupled to protein G Dynabeads (from Invitrogen). HA-tagged and Flag-tagged proteins were immunoprecipitated using anti-HA Affinity matrix (Roche) and Anti-FLAG M2 Affinity Gel (Sigma). Chromatin Binding Assay was performed as previously described [57]. Antibodies used in western blot analysis are anti-HA (3F10; Roche), anti-Flag (M2; Sigma), anti-myc (9E10; Roche), anti-Rpd3 (ab18085; abcam), anti-hexokinase (H2035-01; USBiological), anti SUMO2/3 (Enzo Life Sciences), and anti-histone H3 (ab1791; abcam).

### Protein Expression and Purification

The 6his-T7-Smc5 and 6his-HA-Nse2 proteins were co-expressed in Rosetta 2 (DE3) pLysS cells (Novagen) from pET28a-*SMC5* and pET15b-*HA-MMS21*, respectively. Bacterial cultures were grown at 37°C to A600 = 0.6, before IPTG addition. Cultures were then incubated for 3–4 h at 30°C and harvested by centrifugation. Cell pellets were equilibrated in Lysis Buffer (20% sucrose, 20 mM Tris, 8.0, 1 mM β-mercaptoethanol, 350 mM NaCl, 20 mM Imidazole, 1 mM PMSF, 0.1% IGEPAL), and cells were disrupted by sonication. Cell debris was removed by centrifugation (40,000×g). Hexa-histidine tagged proteins were purified by metal affinity chromatography using Ni-NTA resin (Qiagen) and eluted with 20 mM Tris (pH 8.0), 250 mM NaCl, 1 mM β-mercaptoethanol, and 250 mM imidazole. Fractions containing the Smc5-Mms21 heterodimer were further purified by gel filtration (Superdex 200; GE Healthcare).

### In Vitro Sumoylation Assays

For in vitro sumoylation assays, 100 ODs of *GALp-SMC5* cells that express *SMC5-9myc* from a centromeric vector were shifted to glucose for 5 h, collected and stored at −80°C. After anti-myc immunoprecipitation, reactions were directly performed on complexes immobilized on protein G dynabeads (Invitrogen). Sumoylation was conducted either at 37°C with the human E1, E2, SUMO1, SUMO2, and SUMO3 proteins (Enzo Life Sciences Sumoylation kit, according to the supplier instructions), or at 30°C with recombinant yeast 6 histidine-tagged E1, E2, and Smt3, as previously described [43]. Reactions were run in parallel for wild-type and K75I mutant Smc5/6 complexes, started by addition of ATP, stopped with SDS-PAGE loading buffer, analyzed by western blotting and quantified with Image Lab (Bio-Rad). Since basal sumoylation of wild-type Smc5 is often detectable in the immunoprecipitates, the rate of sumoylation was calculated as the increase in sumoylation divided by the time of incubation in ATP. Small-scale sumoylation reactions of the C-terminal region of Nse4 (residues 246 to 402) were performed in a reaction mixture containing 20 mM HEPES pH 7.5, 5 mM MgCl_2_, 0.1% Tween-20, 100 mM NaCl, 1 mM dithiothreitol, 1 mM ATP, 150 nM hE1, 150 nM hE2, 32 μM hSUMO2, 16 μM Nse4(ct), and 300nM Smc5/Mms21 (wild type or K75I mutant); all proteins were tagged with six histidines, expressed in *E*. *coli* and purified by chromatography on Ni-NTA and gel-filtration columns. Reactions were conducted at 30°C, and samples were taken at different times after ATP addition and stopped with SDS-PAGE loading buffer. SDS-PAGE gels were stained with SYPRO-Ruby (Life Technologies) and the accumulation of Nse4(ct)-SUMO2 was quantified with Image J. Only those time points where the reaction progressed linearly were taken into consideration.

### Scanning Force Microscopy

Smc5-Mms21 or Smc5(K75I)-Mms21 heterodimers were diluted to 30 ng/μl in 50 mM Tris-HCl, pH 7.5, 100 mM NaCl, 2 mM MgCl2 with or without 1 mM ATP and deposited on freshly cleaved mica in the presence of 50 μM spermidine. After 1 min the mica was rinsed with milli Q water and dried with filtered air. Samples were imaged in air by tapping mode SFM using a Nanoscope III or IV (Digital Instruments; Santa Barbara, CA). Silicon tips (NHC-W) with resonance frequency 310–372 kHz were from Nanosensors supplied by Vecco Instruments, Europe. Images were collected at 2 μm × 2 μm, and processed only by flattening to remove background slope. SFM images of Smc5-Mms21 or Smc5(K75I)-Mms21 heterodimers in the absence or in the presence of nucleotide were used for automatic particle detection with custom-made software written in MATLAB. In brief, particles are detected after finding their edges by calculating the gradient of the image intensity at each pixel. The height and area of detected objects was used to calculate a volume in arbitrary pixel units, after subtracting the average background signal of an identical area. Volume units were then normalized using as standard EcRNA polymerase (450 kDa, 678.8 ± 124 measured volume units).

### Coiled Coil Sequence Analysis

Coiled coil and heptad-repeat registry prediction were performed as previously described [58]. For each protein sequence, 14-, 21-, and 28-residue windows were used to plot the coiled coil probability in the upper, middle, and bottom rows, respectively.

### Yeast Strains and Plasmids Used in This Study

**Fig. 1C:** YTR337, YMB2210, pTR1094, pCG2788, pCG2821, pPM2750; **Fig. 1D, E, F:** YMB1840, pTR1094, pCG2788, pCG2821, pPM2750; **Fig. 1G:** YSM2465, pRS315, pTR2395, pTR2400; **Fig. 1H:** YMB1840, YMB1902, pTR1094, pCG2788: **Fig. 2B**: Y557, YMB794, YTR1444, YMB1556; **Fig. 2C**: YMB794, YMB2315, YMB2309; **Fig. 2D**: YMB2315; YMB2309; **Fig. 2E**: YTR337, YTR788, YTR786; **Fig. 2F**: YTR82, YMB1424, YMB1330, YMB1410, YMB1432; **Fig. 2G**: YMB1424, YMB1448, YMB1430, YMB1446, YMB1432, YMB2210; **Fig. 2H**: YMB1446; YMB1432; **Fig. 2I**: YTR854, YMB1117, YMB1345, YMB1120; **Fig. 3A**: YTR31, pTR1094, pTR1621, pNC1828; **Fig. 3B**: YMB1925, YMB1949, YMB1950, YMB1951; **Fig. 3C**: YTR907, pTR1094, pTR1621, pNC1828; **Fig. 3D**: YMB1905, pTR1094, pTR1621; **Fig. 3E:** YTR2373, YMB2214, Y557, YMB1902, pTR1094, pTR1621; **Fig. 3F**: YMB2136, pTR1094, pTR1621, pTR1828; **Fig. 4B**: YMB794, YMB793, YTR1766, YTR1768; **Fig. 4C**: Y557, YTR27, YMB794, YMB793, YTR1766, YTR1768; **Figs.** **4D** and **E**: YPM1812, pTR1094, pTR1621; **Fig. 4F**: YTR3119, YCplac22, pTR1094, pTR1621, pTR3154; **Fig. 5B:** YTR31, YMB1840, pTR1094, pTR1621; **Fig. 5D:** 28S1, pNC2089, pNC2279, p6his-NSE4(ct); **Fig. 6**: 28S1, pNC2089, pNC2279; **Fig. 7B**: YTR907, pTR1094, pTR2158, pTR1967, pTR1969; **Fig. 7C**: YTR31, YMB2136, YCplac22, pTR1094, pTR2158; **Fig. 7D**: YTR29, pTR1094, pTR2158, pTR1967, pTR1969; **Fig. 7E:** Y557, YPM2506; **Fig. 7F:** YMB1840, YMB1902, pTR1094, pTR2158; **Fig. 8A:** Y557, YMB794, YPM2506, YPM2759, YPM2724; **Fig. 8B:** YMB794, YPM2506, YPM2759, YPM2724; **S2A Fig.**: YTR622, YMB628; **S2B Fig.**: Y557, Y570, YMB1840, pCG2788, YTR622, YTR628, YTR506, YTR3135; **S2C Fig.**: YTR628; **S3 Fig.**: Y557, YTR1435, YMB1452, YMB1454, YMB1456; **S4 Fig.**: YTR907, pTR1094, pTR1621; **S5 Fig.**: YMB1840, pTR1094, pTR1621; **S6 and S7 Figs.**: 28S1, pNC2089, pNC2279, pNC2094.

## Supporting Information

S1 Data Excel spreadsheet containing, in separate sheets, the underlying numerical data for Fig. 1E, Fig. 4H, Fig. 4J, Fig. 5, Fig. 6E, and S2B Fig.
(XLSX)

S1 Fig Related to Fig. 1. Molecular view of the Smc5-Mms21 interaction surfaces. Mms21 is shown in yellow, while the coiled coil of Smc5 is shown in gray. The side chains of residues mutated in *smc5-S1*, *smc5-S2* and *smc5-S3* are shown as sticks. Note that all mutated residues are directly facing the Mms21 protein.
(TIF)

S2 Fig Related to Fig. 1. Chromosome disjunction and segregation defects in *mms21Δc* mutant cells after DNA damage. **A**. Wild-type (wt) and *mms21Δc* cells were arrested in G1 at 30°C with alpha factor. Arrested cells were transitorily (30 min) treated with 0.01% MMS before release into a synchronous cell cycle. Samples were taken at the indicated times and processed for FACS analysis and pulse field gel electrophoresis (PFGE). Note that both cultures enter S phase and reach 2C DNA content with similar kinetics. PFGE shows chromosome bands of reduced intensity at time points 20 and 40 min, as replicating chromosomes from wild-type cells remain in the well and fail to enter into the gel; as expected, bands double the intensity after completion of S phase (60 min onwards). In contrast, chromosomes from *mms21Δc* cells display a non-disjunction phenotype, evidenced by a failure to double in intensity after S phase. **B**. Analysis of nuclear and specific chromosomal marker segregation. Wild-type and *mms21Δc* cells were treated as in A; *smc5-S1* cells were treated as depicted in Fig. 1E. Nuclear segregation was scored after staining with Hoechst; Centromere 3 (CEN3) was labeled with a battery of lac operators in cells that also express a lacI-GFP fusion. The telomeric flank of the rDNA array (tetO:487) was labeled with a battery of tet operators in cells that express a tetR-YFP fusion. Nuclear segregation/missegregation was scored in all large budded cells entering a second cell cycle (rebudding). CEN3 and rDNA segregation was scored in all binucleated cells. Note that one out of four *mms21Δc* cells fail to segregate the nucleus; a more detailed analysis of individual loci segregation indicates that one out of three *mms21Δc* cells fail to separate chromosome 3, and almost all of them fail to segregate the rDNA array. In contrast, *smc5-S1* cells have a more drastic effect in chromosome segregation, probably because of impairment of the Mms21-dependent sumoylation and elimination of the essential Smc5-Mms21 interaction. **C**. Examples of *mms21Δc* cells at time point 140 min displaying rDNA (top) or nuclear (bottom) segregation defects.
(TIF)

S3 Fig Related to Fig. 2. Smc5 sumoylation requires the Nse4, Nse5, and Nse6 subunits of the Smc5/6 complex. **A**. Auxin-induced degrons (aid) of Nse4, Nse5, or Nse6 display severe growth defects. Serial dilutions of wild-type, *nse4-aid*, *nse5-aid*, and *nse6-aid* cells were spotted on YPD plates or YPD plates containing 1 mM of Indole-3-Acetic Acid (IAA). Note that all degron mutants are sensitive to IAA. **B**. Smc5 sumoylation depends on Nse4-6 subunits. Exponentially growing cultures of the indicated strains were treated with 1 mM auxin for 2 h to induce degradation of the degron-fused proteins. Samples were processed for pull-down analysis as in Fig. 2B.
(TIF)

S4 Fig Related to Fig. 3. The ATPase Smc5(K75I) mutant protein efficiently competes with wild-type Smc5 for binding to chromatin. Chromatin fractionation assay from wild-type cells expressing an ectopic 9myc-tagged copy of the indicated *SMC5* alleles. Controls for chromatin-bound (histone H3), nuclear soluble (Rpd3), and cytoplasmic soluble (Hexokinase; Hxk) proteins are shown; WCE: Whole Cell Extract; SN: Supernatant; Chr: Chromatin fraction.
(TIF)

S5 Fig Related to Fig. 5. In vitro sumoylation reactions on immunoprecipitated Smc5-9myc. Smc5/6 complexes immunopurified and bound on dynabeads were incubated with the yeast E1, E2 and SUMO proteins at 30°C for 1 h, as described in Materials and Methods. Reactions were stopped by addition of SDS-PAGE loading buffer and analyzed by SDS-PAGE and immunoblotting with anti-myc. Note that sumoylation can be detected for wild-type Smc5, but not the ATPase-defective smc5(K75I) mutant protein.
(TIF)

S6 Fig Related to Fig. 5 and 6. Expression and purification of Smc5-Mms21 heterodimers. **A**. Smc5 was expressed alone or in combination with Mms21 in Rosetta 2 (DE3) pLysS cells. Lysates (L) were incubated with NiNTA beads and eluted with imidazole to purify Smc5. WCE: Whole Cell Extract; FT: Flow through; E: Eluate. **B**. Same as in A, but Smc5 was co-expressed with either the wild-type Mms21 protein or a double Mms21-M1,M2 mutant protein that cannot bind Smc5 [24]. Note that Smc5 is expressed at very low levels when Mms21 is not co-expressed or cannot interact with Smc5. **C**. Wild type (wt) or K75I (KI) mutant was co-expressed with Mms21 in Rosetta 2 (DE3) pLysS cells. Following NiNTA purification, the Smc5-Mms21 heterodimer was further purified by gel filtration (S200: Superdex 200; GE Healthcare). Samples were run on an SDS-PAGE gel and stained with coomasie.
(TIF)

S7 Fig Related to Fig. 6. High-throughput SFM image analysis: estimation of particle volume and height. SFM image data of SMC5-Mms21 heterodimer in the absence or in the presence of ATP were collected as described in the main text. **A**. An example image of Smc5-Mms21 heterodimer in the absence of ATP. **B**. Particles were automatically detected in the SFM image by Sobel edge detection, which calculates a gradient of intensity at each pixel, and defined by red contours. Particle volumes were subsequently calculated by adding the volume of each pixel, defined as pixel area multiplied by height minus average background height, within the detected contours. The volume distribution was based on all detected particles. Scale bar 100 nm. Height is indicated by color as shown in the inserted bar at the right upper corner.
(TIF)

S8 Fig Related to Fig. 7. Coiled coil probability in Smc5 mutants with altered coiled coil sequence. **A**. Coiled coil probability in the different mutant studied in Fig. 6. 14-, 21-, and 28-residue windows are used in upper, middle, and bottom row; numerical values are colored as shown in the legend. Yellow rectangle marks Mms21 docking site. Ruler below marks amino acid position in sequence. **B**. Prediction of the heptad repeat pattern around the P393 position for the *SMC5* wild type and the *smc5-DLEL* mutant. Note that the *DLEL* mutation allows recovery of the heptad repeat pattern by placing charged residues in positions 5 and 7, and a hydrophobic residue (L) in position 1 of the following repeat.
(TIF)

S1 Table Relevant genotype of yeast strains and plasmids used in this study. (DOCX)

## Abbreviations:

aid: Auxin-induced degron
CC1: first coiled coil
CEN3: Centromere 3
Chr: Chromatin fraction
DAPI: 4’,6-diamidino-2-phenylindole
FACS: Fluorescence-Activated Cell Sorting
HF: 6xHis-Flag
IAA: Indole-3-Acetic Acid
MMS: methyl methanesulfonate
NBD: nucleotide binding domain
P.D.: pull down
PFGE: pulse field gel electrophoresis
rDNA: ribosomal DNA
SFM: scanning force microscopy
SMC: Structural Maintenance of Chromosomes
SN: Supernatant
WCE: Whole Cell Extract
