# Supplementary figures and images for: ATPase-Dependent Control of the Mms21 SUMO Ligase during DNA Repair
Source: PLoS Biol. 2015 Mar 12;13(3):e1002089. doi: 10.1371/journal.pbio.1002089 (PMC4357442; doi:10.1371/journal.pbio.1002089)

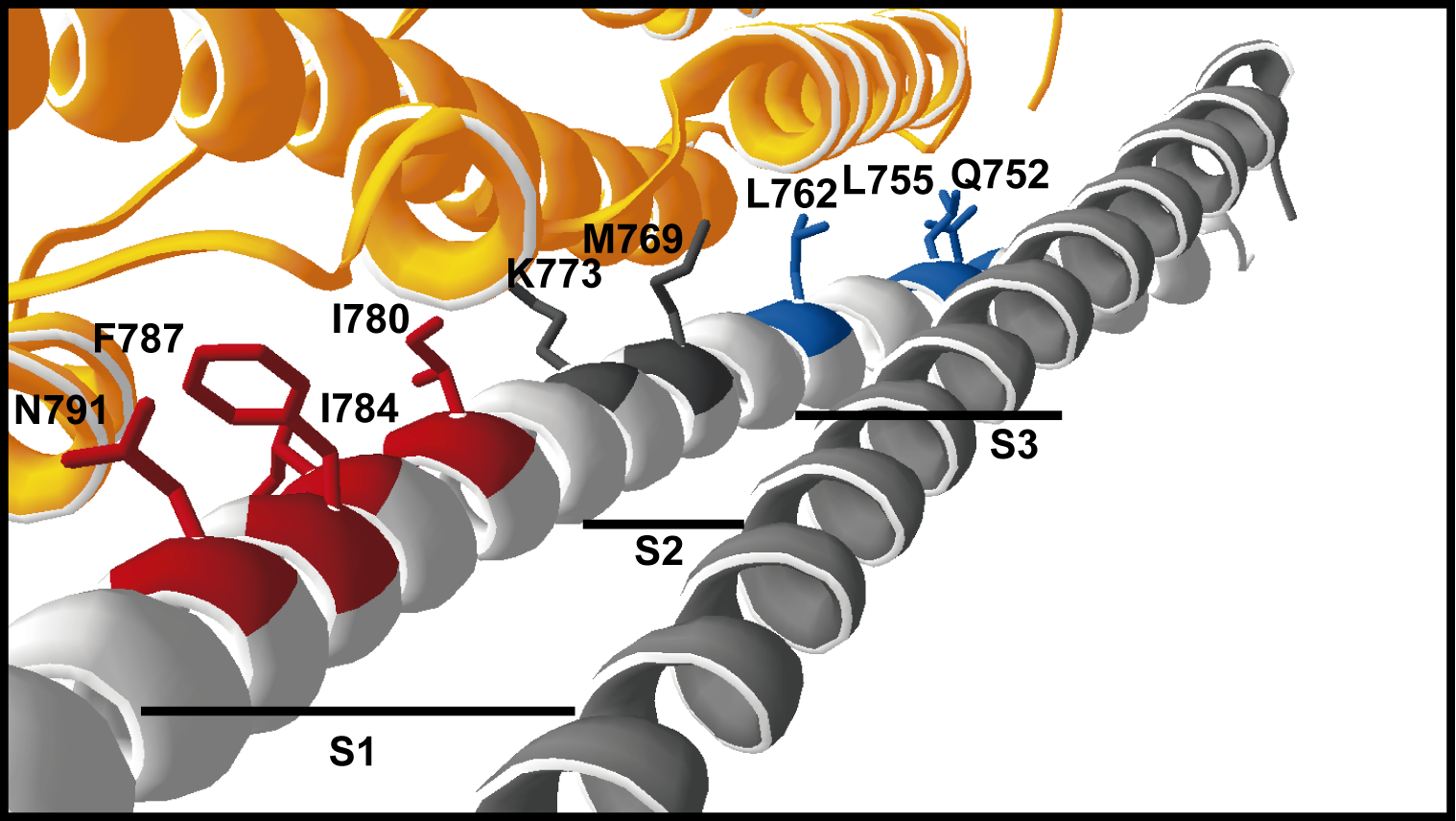

Supplement: S1 Fig — Mms21 is shown in yellow, while the coiled coil of Smc5 is shown in gray. The side chains of residues mutated in smc5-S1, smc5-S2 and smc5-S3 are shown as sticks. Note that all mutated residues are directly facing the Mms21 protein. (TIF) [file pbio.1002089.s002.tif]

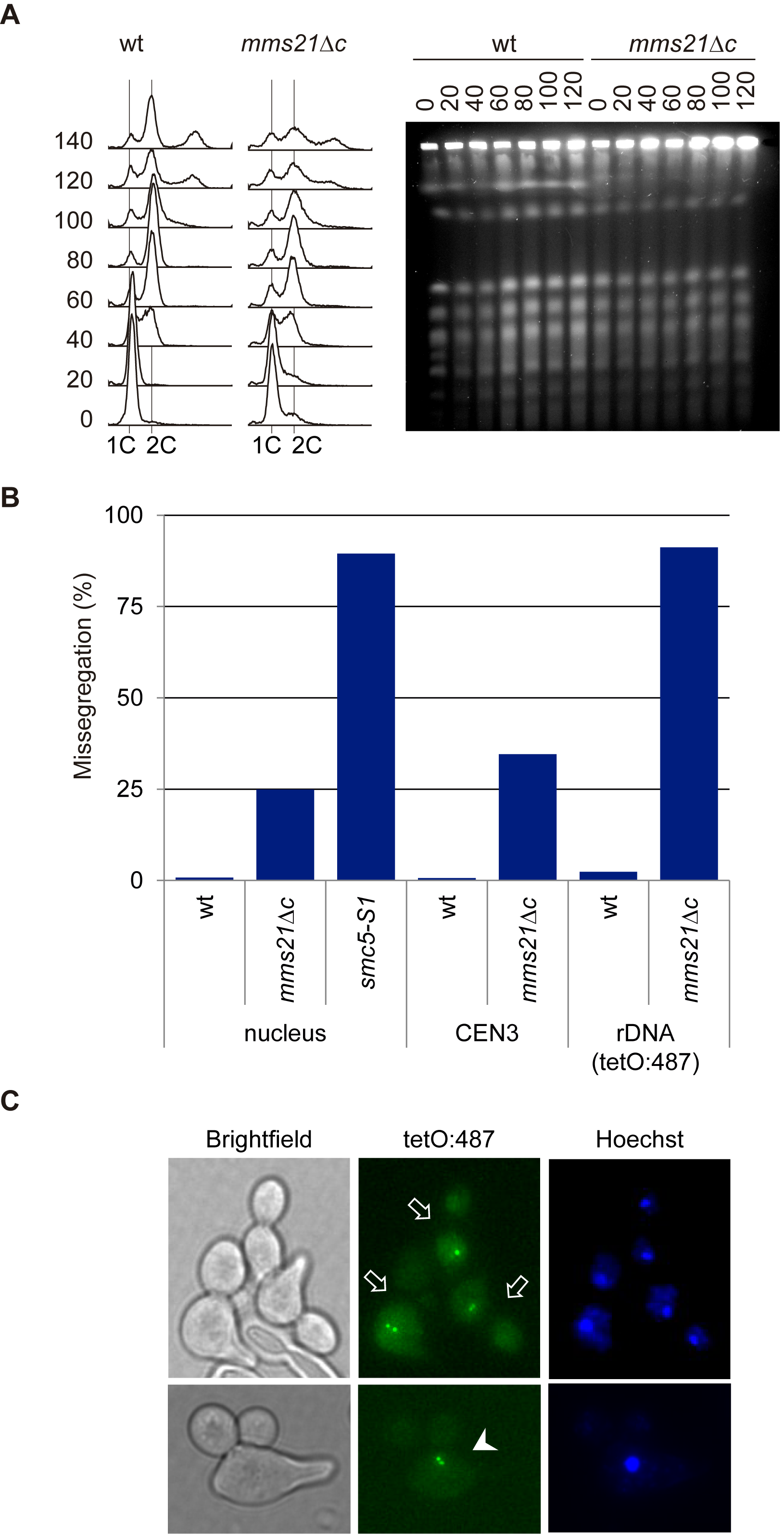

Supplement: S2 Fig — A. Wild-type (wt) and mms21Δc cells were arrested in G1 at 30°C with alpha factor. Arrested cells were transitorily (30 min) treated with 0.01% MMS before release into a synchronous cell cycle. Samples were taken at the indicated times and processed for FACS analysis and pulse field gel electrophoresis (PFGE). Note that both cultures enter S phase and reach 2C DNA content with similar kinetics. PFGE shows chromosome bands of reduced intensity at time points 20 and 40 min, as replicating chromosomes from wild-type cells remain in the well and fail to enter into the gel; as expected, bands double the intensity after completion of S phase (60 min onwards). In contrast, chromosomes from mms21Δc cells display a non-disjunction phenotype, evidenced by a failure to double in intensity after S phase. B. Analysis of nuclear and specific chromosomal marker segregation. Wild-type and mms21Δc cells were treated as in A; smc5-S1 cells were treated as depicted in Fig. 1E. Nuclear segregation was scored after staining with Hoechst; Centromere 3 (CEN3) was labeled with a battery of lac operators in cells that also express a lacI-GFP fusion. The telomeric flank of the rDNA array (tetO:487) was labeled with a battery of tet operators in cells that express a tetR-YFP fusion. Nuclear segregation/missegregation was scored in all large budded cells entering a second cell cycle (rebudding). CEN3 and rDNA segregation was scored in all binucleated cells. Note that one out of four mms21Δc cells fail to segregate the nucleus; a more detailed analysis of individual loci segregation indicates that one out of three mms21Δc cells fail to separate chromosome 3, and almost all of them fail to segregate the rDNA array. In contrast, smc5-S1 cells have a more drastic effect in chromosome segregation, probably because of impairment of the Mms21-dependent sumoylation and elimination of the essential Smc5-Mms21 interaction. C. Examples of mms21Δc cells at time point 140 min displaying rDNA (top) or nucle [file pbio.1002089.s003.tif]

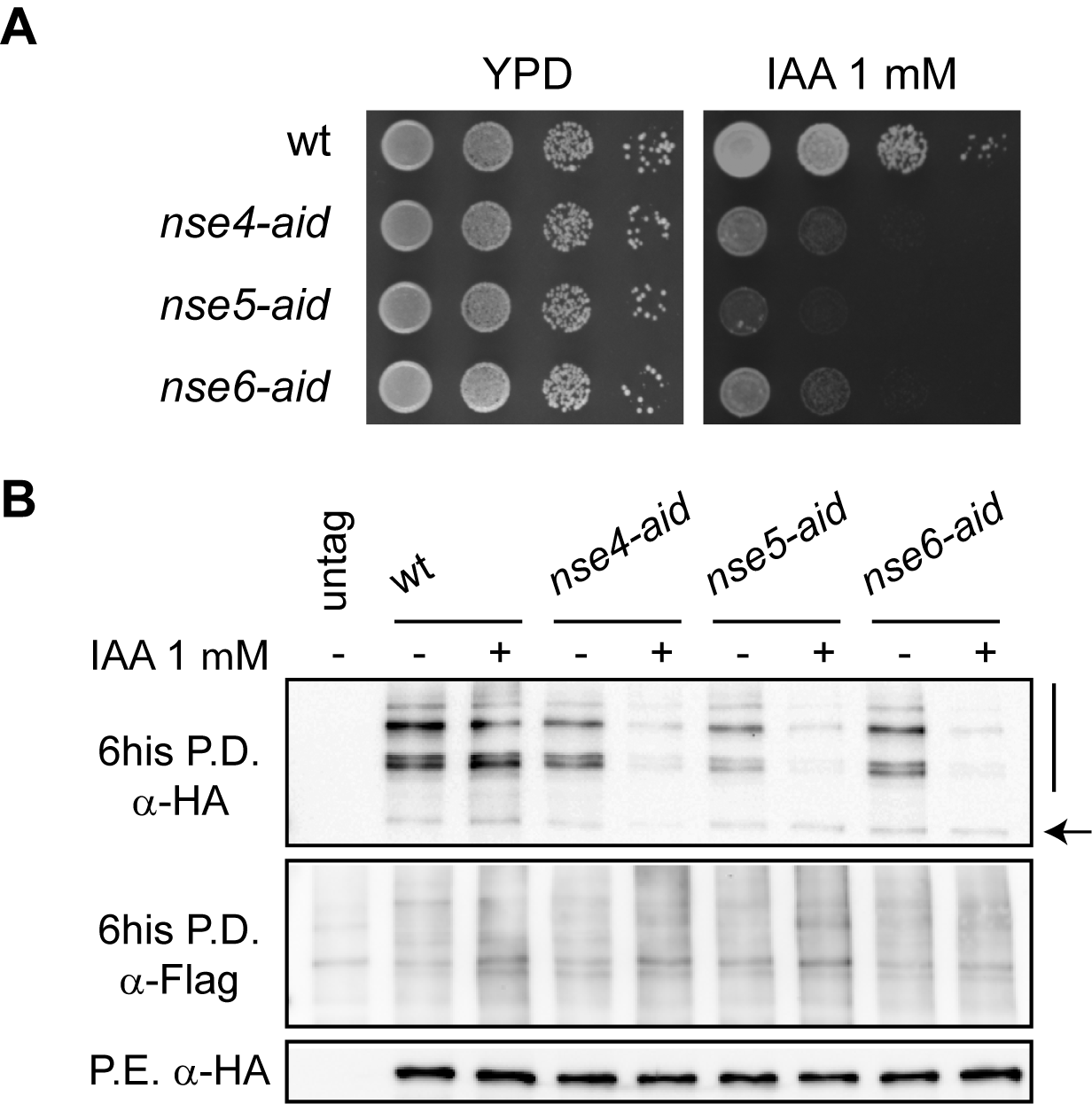

Supplement: S3 Fig — A. Auxin-induced degrons (aid) of Nse4, Nse5, or Nse6 display severe growth defects. Serial dilutions of wild-type, nse4-aid, nse5-aid, and nse6-aid cells were spotted on YPD plates or YPD plates containing 1 mM of Indole-3-Acetic Acid (IAA). Note that all degron mutants are sensitive to IAA. B. Smc5 sumoylation depends on Nse4-6 subunits. Exponentially growing cultures of the indicated strains were treated with 1 mM auxin for 2 h to induce degradation of the degron-fused proteins. Samples were processed for pull-down analysis as in Fig. 2B. (TIF) [file pbio.1002089.s004.tif]

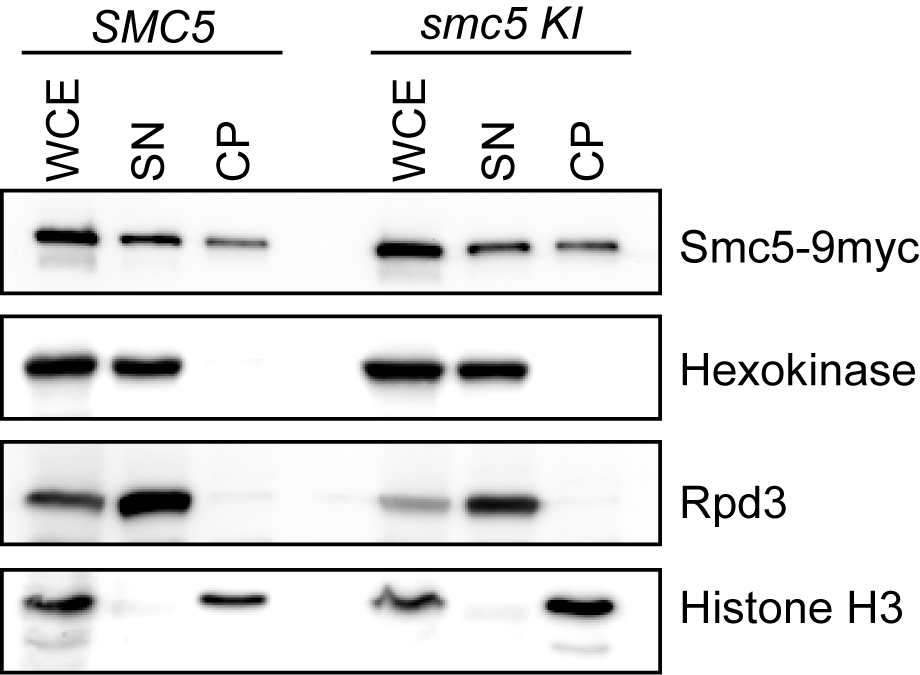

Supplement: S4 Fig — Chromatin fractionation assay from wild-type cells expressing an ectopic 9myc-tagged copy of the indicated SMC5 alleles. Controls for chromatin-bound (histone H3), nuclear soluble (Rpd3), and cytoplasmic soluble (Hexokinase; Hxk) proteins are shown; WCE: Whole Cell Extract; SN: Supernatant; Chr: Chromatin fraction. (TIF) [file pbio.1002089.s005.tif]

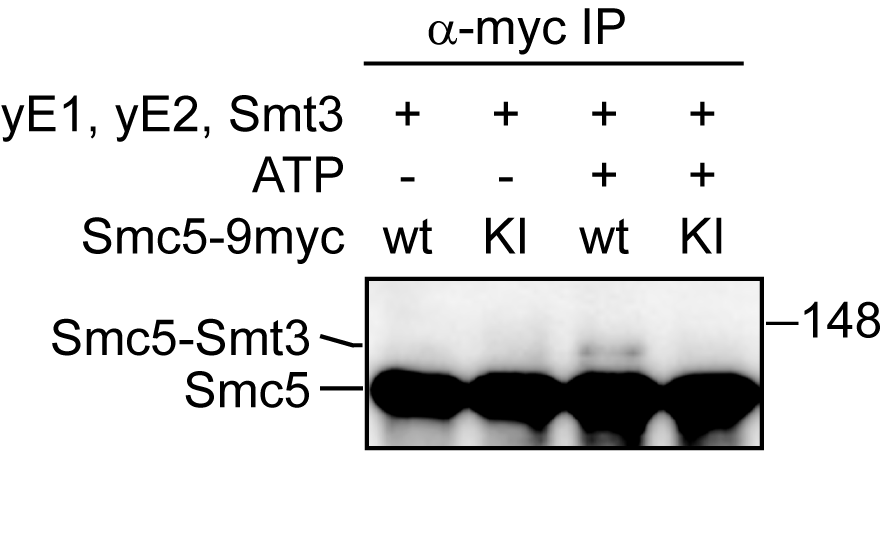

Supplement: S5 Fig — Smc5/6 complexes immunopurified and bound on dynabeads were incubated with the yeast E1, E2 and SUMO proteins at 30°C for 1 h, as described in Materials and Methods. Reactions were stopped by addition of SDS-PAGE loading buffer and analyzed by SDS-PAGE and immunoblotting with anti-myc. Note that sumoylation can be detected for wild-type Smc5, but not the ATPase-defective smc5(K75I) mutant protein. (TIF) [file pbio.1002089.s006.tif]

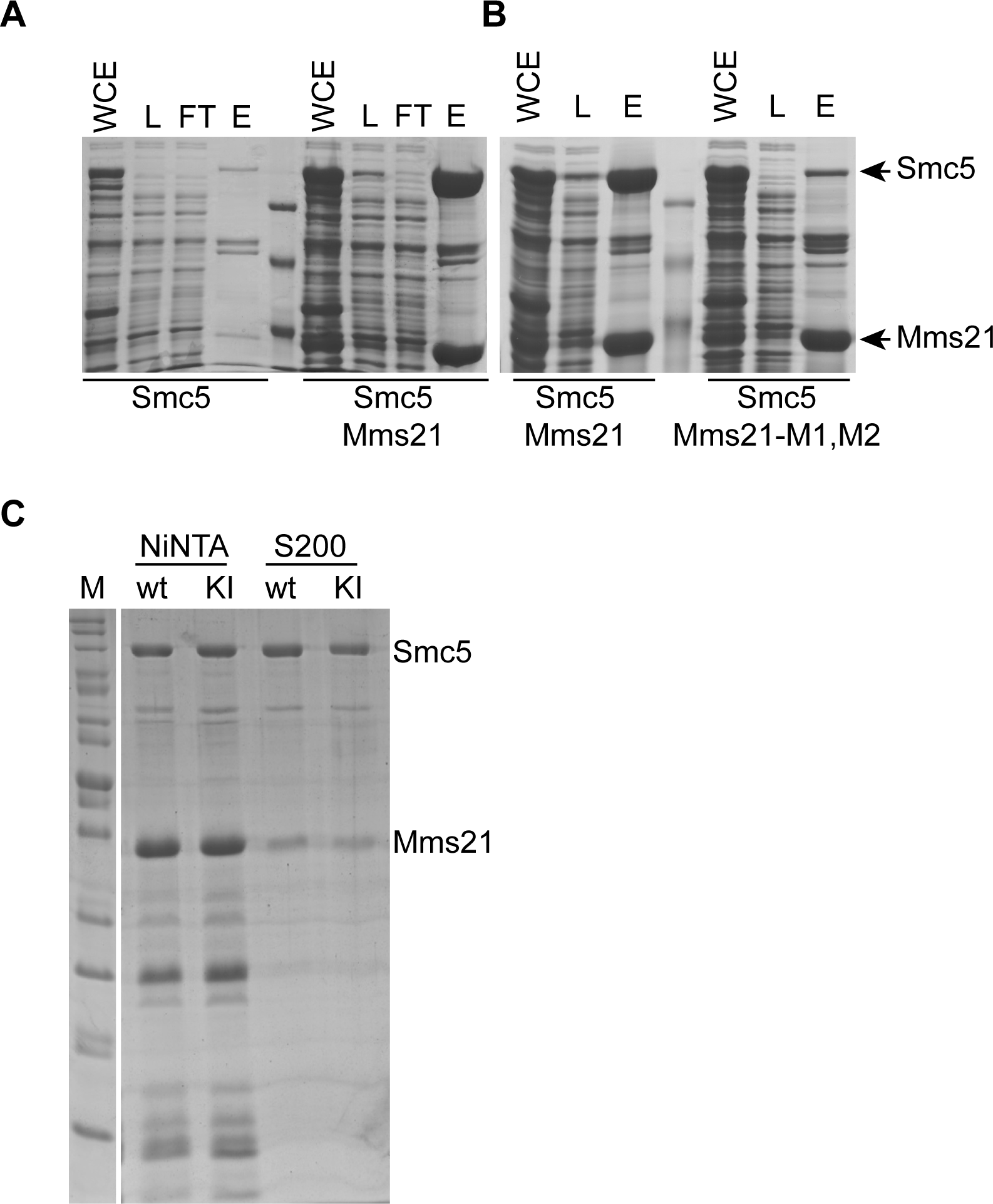

Supplement: S6 Fig — A. Smc5 was expressed alone or in combination with Mms21 in Rosetta 2 (DE3) pLysS cells. Lysates (L) were incubated with NiNTA beads and eluted with imidazole to purify Smc5. WCE: Whole Cell Extract; FT: Flow through; E: Eluate. B. Same as in A, but Smc5 was co-expressed with either the wild-type Mms21 protein or a double Mms21-M1,M2 mutant protein that cannot bind Smc5 [24]. Note that Smc5 is expressed at very low levels when Mms21 is not co-expressed or cannot interact with Smc5. C. Wild type (wt) or K75I (KI) mutant was co-expressed with Mms21 in Rosetta 2 (DE3) pLysS cells. Following NiNTA purification, the Smc5-Mms21 heterodimer was further purified by gel filtration (S200: Superdex 200; GE Healthcare). Samples were run on an SDS-PAGE gel and stained with coomasie. (TIF) [file pbio.1002089.s007.tif]

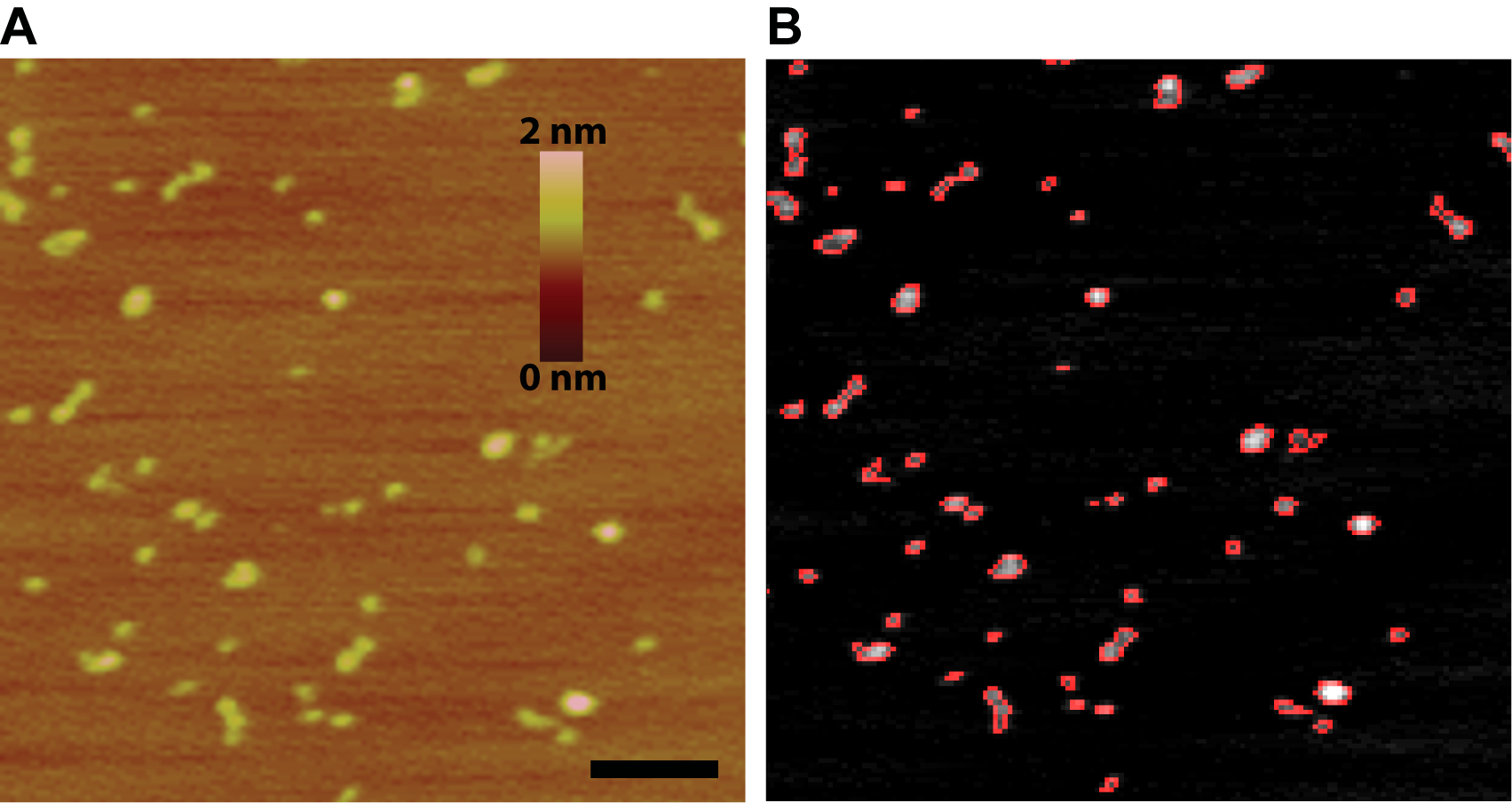

Supplement: S7 Fig — SFM image data of SMC5-Mms21 heterodimer in the absence or in the presence of ATP were collected as described in the main text. A. An example image of Smc5-Mms21 heterodimer in the absence of ATP. B. Particles were automatically detected in the SFM image by Sobel edge detection, which calculates a gradient of intensity at each pixel, and defined by red contours. Particle volumes were subsequently calculated by adding the volume of each pixel, defined as pixel area multiplied by height minus average background height, within the detected contours. The volume distribution was based on all detected particles. Scale bar 100 nm. Height is indicated by color as shown in the inserted bar at the right upper corner. (TIF) [file pbio.1002089.s008.tif]

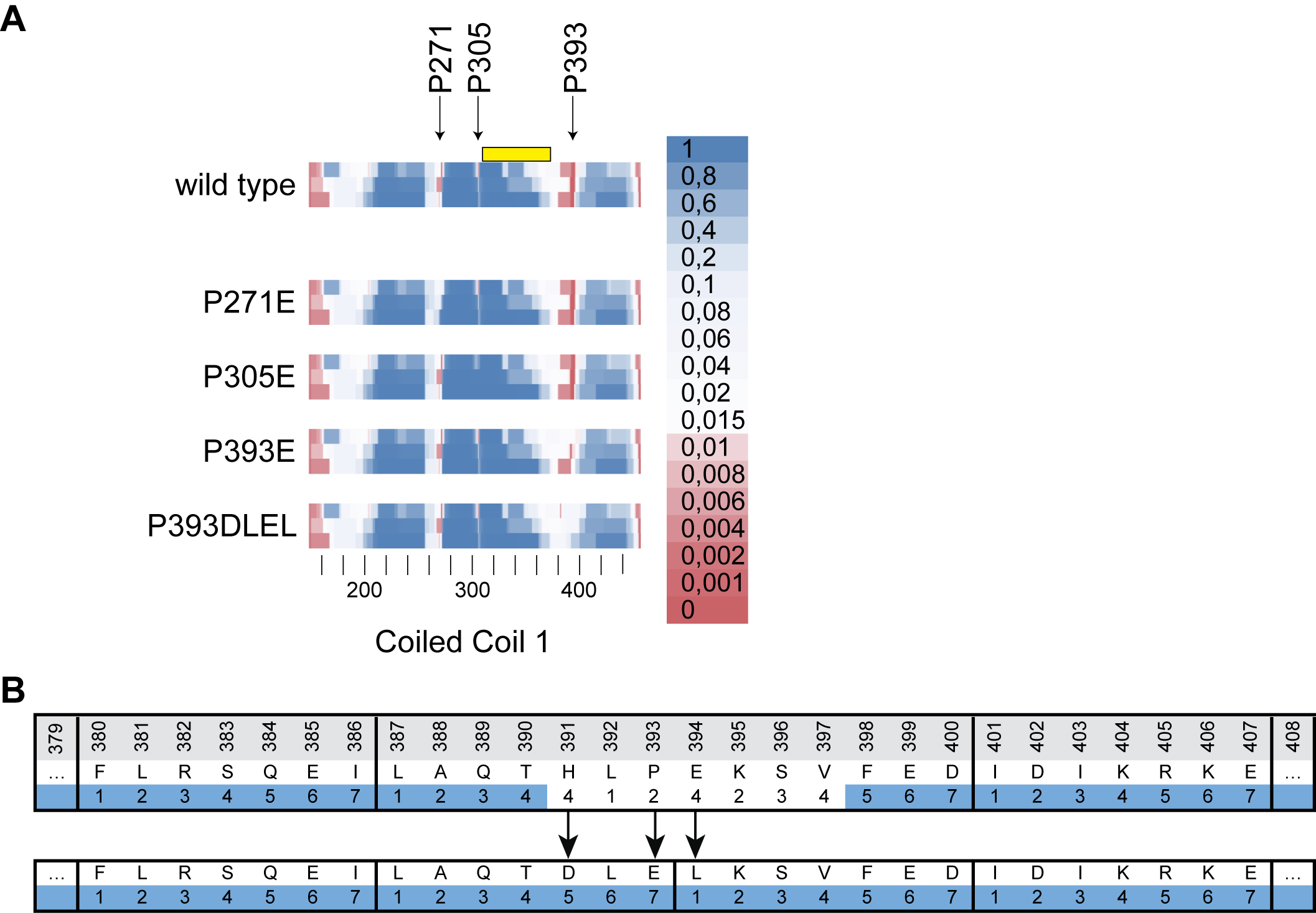

Supplement: S8 Fig — A. Coiled coil probability in the different mutant studied in Fig. 6. 14-, 21-, and 28-residue windows are used in upper, middle, and bottom row; numerical values are colored as shown in the legend. Yellow rectangle marks Mms21 docking site. Ruler below marks amino acid position in sequence. B. Prediction of the heptad repeat pattern around the P393 position for the SMC5 wild type and the smc5-DLEL mutant. Note that the DLEL mutation allows recovery of the heptad repeat pattern by placing charged residues in positions 5 and 7, and a hydrophobic residue (L) in position 1 of the following repeat. (TIF) [file pbio.1002089.s009.tif]
