# Supplementary material for: ATPase-Dependent Control of the Mms21 SUMO Ligase during DNA Repair
Source: PLoS Biol. 2015 Mar 12;13(3):e1002089. doi: 10.1371/journal.pbio.1002089 (PMC4357442; doi:10.1371/journal.pbio.1002089)
Supplement: S1 Table — (DOCX) [file pbio.1002089.s010.docx]

**S1 Table.** Relevant genotype of yeast strains and plasmids used in this study.

| **Yeast Strains** | |
| --- | --- |
| YTR27 | *MATa his3Δ200 leu2Δ0 met15Δ0 trpΔ63 ura3Δ0 SMC5-9myc:TRP* |
| YTR29 | *MATa his3Δ200 leu2Δ0 met15Δ0 trpΔ63 ura3Δ0 KanMX:GAL-3HA-SMC5* |
| YTR31 | *MATa his3Δ200 leu2Δ0 met15Δ0 trpΔ63 ura3Δ0 KanMX:GAL-3HA-SMC5 bar1::URAca* |
| YTR82 | *MATa bar1Δ leu2-3,112 ura3-52 his3-Δ200 trp1-Δ63 ade2-1 lys2-801 pep4 NSE3-9myc:TRP* |
| YTR337 | *MATa bar1Δ leu2-3,112 ura3-52 his3-Δ200 trp1-Δ63 ade2-1 lys2-801 pep4* |
| YTR506 | *MATa bar1Δleu2-3,112 ura3-52 his3-Δ200 trp1-Δ63 ade2-1 lys2-801 pep4 his3::pHIS3-lacI-NLS-GFP:HIS3 CEN3:256xlacO:URA3* |
| Y557 | *MATa his3Δ1 leu2Δ0 met15Δ0 ura3Δ0 6his-Flag-SMT3:kanMX6* |
| Y570 | *MATa his3Δ1 leu2Δ0 met15Δ0 ura3Δ0 6his-Flag-SMT3:KANMX6 mms21Δc:hphMX4* |
| YTR622 | *MATa bar1Δ leu2-3,112 ura3-52 his3-Δ200 trp1-Δ63 ade2-1 lys2-801 pep4 TetR-YFP:ADE2 tetO(5.6Kb):487Kb ChrXII HIS3* |
| YMB628 | *MATa bar1Δ leu2-3,112 ura3-52 his3-Δ200 trp1-Δ63 ade2-1 lys2-801 pep4 TetR-YFP:ADE2 tetO(5.6Kb):487Kb ChrXII HIS3 mms21Δc:hphMX4* |
| YTR786 | *MATa bar1Δ leu2-3,112 ura3-52 his3-Δ200 trp1-Δ63 ade2-1 lys2-801 pep4 nse5-2-9myc:TRP* |
| YTR788 | *MATa bar1Δ leu2-3,112 ura3-52 his3-Δ200 trp1-Δ63 ade2-1 lys2-801 pep4 nse3-2-9myc:TRP* |
| YMB793 | *MATa his3Δ1 leu2Δ0 met15Δ0 ura3Δ0 6his-Flag-SMT3:KANMX6 nse2ΔC::hphMX4 SMC5-9myc:HIS* |
| YMB794 | *MATa his3Δ1 leu2Δ0 met15Δ0 ura3Δ0 6his-Flag-SMT3:KANMX6 SMC5-9myc:HIS* |
| YTR854 | *MATa bar1Δ leu2-3,112 ura3-52 his3-Δ200 trp1-Δ63 ade2-1 lys2-801 pep4 NSE3-9myc:TRP 6his-Flag-SMT3:Kan* |
| YTR907 | *MATa ade2-1 trp1Δ2 can1-100 leu2-3,112 his3-11,15 ura3-52 6his-Flag-SMT3:KanMX4* |
| YMB1117 | *MATa bar1Δ leu2-3,112 ura3-52 his3-Δ200 trp1-Δ63 ade2-1 lys2-801 pep4 SMC5-6HA:HIS 6his-Flag-SMT3:Kan* |
| YMB1120 | *MATa bar1Δ leu2-3,112 ura3-52 his3-Δ200 trp1-Δ63 ade2-1 lys2-801 pep4 nse5-2-9myc:TRP SMC5-6HA:HIS 6his-Flag-SMT3:Kan* |
| YMB1330 | *MATa bar1Δ leu2-3,112 ura3-52 his3-Δ200 trp1-Δ63 ade2-1 lys2-801 pep4 nse3-2-9myc:TRP SMC5-6Flag:KanMX* |
| YMB1345 | *MATa bar1Δ leu2-3,112 ura3-52 his3-Δ200 trp1-Δ63 ade2-1 lys2-801 pep4 nse3-2-9myc:TRP 6his-Flag-SMT3:Kan SMC5-6HA:hphNT* |
| YMB1410 | *MATa bar1Δ leu2-3,112 ura3-52 his3-Δ200 trp1-Δ63 ade2-1 lys2-801 pep4 NSE5-9myc:TRP SMC5-6Flag:KanMX* |
| YMB1424 | *MATa bar1Δ leu2-3,112 ura3-52 his3-Δ200 trp1-Δ63 ade2-1 lys2-801 pep4 NSE3-9myc:TRP SMC5-6Flag:KanMX* |
| YMB1430 | *MATa bar1Δ leu2-3,112 ura3-52 his3-Δ200 trp1-Δ63 ade2-1 lys2-801 pep4 nse3-2-9myc:TRP SMC5-6Flag:KanMX MMS21-6HA:HIS* |
| YMB1432 | *MATa bar1Δ leu2-3,112 ura3-52 his3-Δ200 trp1-Δ63 ade2-1 lys2-801 pep4 nse5-2-9myc:TRP SMC5-6Flag:KanMX MMS21-6HA:HIS* |
| YTR1435 | *MATa bar1Δ leu2-3,112 ura3-52 his3-Δ200 trp1-Δ63 ade2-1 lys2-801 pep4 SMC5-6HA:HIS 6his-Flag-SMT3:hph ura3-1::ADH1-OsTIR1-9myc:URA3* |
| YTR1444 | *MATa his3Δ200 leu2Δ0 met15Δ0 trpΔ63 ura3Δ0 GAL-3HA-SMC6:HIS bar1::URAca 6his-Flag-SMT3:KanMX4 SMC5-9myc:TRP* |
| YMB1446 | *MATa bar1Δ leu2-3,112 ura3-52 his3-Δ200 trp1-Δ63 ade2-1 lys2-801 pep4 NSE5-9myc:TRP SMC5-6Flag:KanMX MMS21-6HA:HIS* |
| YMB1448 | *MATa bar1Δ leu2-3,112 ura3-52 his3-Δ200 trp1-Δ63 ade2-1 lys2-801 pep4 NSE3-9myc:TRP SMC5-6Flag:KanMX MMS21-6HA:HIS* |
| YMB1452 | *MATa bar1Δ leu2-3,112 ura3-52 his3-Δ200 trp1-Δ63 ade2-1 lys2-801 pep4 SMC5-6HA:HIS 6his-Flag-SMT3:hph ura3-1::ADH1-OsTIR1-9myc:URA3 nse4-aid:kanMX* |
| YMB1454 | *MATa bar1Δ leu2-3,112 ura3-52 his3-Δ200 trp1-Δ63 ade2-1 lys2-801 pep4 SMC5-6HA:HIS 6his-Flag-SMT3:hph ura3-1::ADH1-OsTIR1-9myc:URA3 nse5-aid:kanMX* |
| YMB1456 | *MATa bar1Δ leu2-3,112 ura3-52 his3-Δ200 trp1-Δ63 ade2-1 lys2-801 pep4 SMC5-6HA:HIS 6his-Flag-SMT3:hph ura3-1::ADH1-OsTIR1-9myc:URA3 nse6-aid:kanMX* |
| YMB1556 | *MATa his3Δ200 leu2Δ0 met15Δ0 trpΔ63 ura3Δ0 GAL-3HA-SMC6:HIS bar1::URAca 6his-Flag-SMT3:KanMX4 SMC5-9myc:TRP pRS415-smc6-1* |
| YTR1766 | *MATa his3Δ1 leu2Δ0 met15Δ0 ura3Δ0 6his-Flag-SMT3:KANMX6 SMC5-9myc:HIS MMS21-3HA-UBC9:hphNT* |
| YTR1768 | *MATa his3Δ1 leu2Δ0 met15Δ0 ura3Δ0 6his-Flag-SMT3:KANMX6 SMC5-9myc:HIS mms21ΔC-3HA-UBC9:hphNT* |
| YPM1812 | *MATa ade2-1 trp1Δ2 can1-100 leu2-3,112 his3-11,15 ura3-52 6his-Flag-SMT3:KanMX4 MMS21-3HA-UBC9:hphNT* |
| YMB1840 | *MATa his3Δ200 leu2Δ0 met15Δ0 trpΔ63 ura3Δ0 KanMX:GAL-3HA-SMC5 bar1::URAca 6His-Flag-SMT3:hphNT* |
| YMB1852 | *MATa ade2-1 ura3-1 his3-11,15 trp1-1 leu2-3,112 can1-100 ura3-1::ADH1-OsTIR1-9myc:URA3 6his-Flag-SMT3:hphMX* |
| YMB1902 | *MATa his3Δ200 leu2Δ0 met15Δ0 trpΔ63 ura3Δ0 KanMX:GAL-3HA-SMC5 bar1::URAca 6His-Flag-SMT3:hphNT SMC1-6HA:natNT* |
| YMB1905 | *MATa his3Δ200 leu2Δ0 met15Δ0 trpΔ63 ura3Δ0 kan:GAL-3HA-SMC5 bar1::URAca 6his-Flag-SMT3:hphNT NSE4-6HA:natNT* |
| YMB1925 | *MATa ade2-1 trp1Δ2 can1-100 leu2-3,112 his3-11,15 ura3-52 6his-Flag-SMT3:KanMX4 pADH1p-SMC5:9myc* |
| YMB1949 | *MATa ade2-1 trp1Δ2 can1-100 leu2-3,112 his3-11,15 ura3-52 6his-Flag-SMT3:KanMX4 pADH1p-SMC5(K75I):9myc* |
| YMB1950 | *MATa bar1Δ leu2-3,112 ura3-52 his3-Δ200 trp1-Δ63 ade2-1 lys2-801 pep4 MMS21-3HA:HIS pADH1p-SMC5:9myc* |
| YMB1951 | *MATa bar1Δ leu2-3,112 ura3-52 his3-Δ200 trp1-Δ63 ade2-1 lys2-801 pep4 MMS21-3HA:HIS pADH1p-SMC5(K75I):9myc* |
| YMB2136 | *MATa his3Δ200 leu2Δ0 met15Δ0 trp1Δ63 ura3D0 GAL-3HA-SMC5:kanMX bar1::URAca MMS21-6HA:natNT* |
| YMB2210 | *MATa bar1Δ leu2-3,112 ura3-52 his3-Δ200 trp1-Δ63 ade2-1 lys2-801 pep4 MMS21-6HA:natNT* |
| YMB2214 | *MATa his3Δ1 leu2Δ0 met15Δ0 ura3Δ0 6His-FLAG-SMT3:kanMX6 SMC1-6HA:natNT* |
| YMB2309 | *MATa his3Δ200 leu2Δ0 met15Δ0 trpΔ63 ura3Δ0 HIS:GAL-3HA-SMC6 bar1::URAca 6his-Flag-SMT3:KanMX4 SMC5-9myc:TRP pRS415-smc6-1 MMS21-6HA:natNT* |
| YMB2315 | *MATa his3Δ1 leu2Δ0 met15Δ0 ura3Δ0 6his-Flag-SMT3:KANMX6 SMC5-9myc:HIS MMS21-6HA:natNT* |
| YTR2373 | *MATa his3Δ1 leu2Δ0 met15Δ0 ura3Δ0 6His-FLAG-SMT3:kanMX4 mms21Δc:hphMX4 SMC1-6HA:natNT* |
| YSM2465 | *MATa his3Δ1 leu2Δ0 met15Δ0 ura3Δ0 6HisFLAG-smt3:kanMX4 GALS-3HA-NSE2:natNT2 SMC1-6HA:HIS3* |
| YPM2506 | *MATa his3Δ1 leu2Δ0 met15Δ0 ura3Δ0 6HisFLAG-smt3::kanMX4 SMC5(P393DLEL)-9Myc:natNT* |
| YPM2724 | *MATa his3Δ1 leu2Δ0 met15Δ0 ura3Δ0 6HisFLAG-smt3::kanMX4 SMC5-9Myc:natNT MMS21-3HA-UBC9:hphNT* |
| YPM2759 | *MATa his3Δ1 leu2Δ0 met15Δ0 ura3Δ0 6HisFLAG-smt3::kanMX4 SMC5(P393DLEL)-9Myc:natNT MMS21-3HA-UBC9:hphNT* |
| YTR3119 | *MATa his3Δ200 leu2Δ0 met15Δ0 trpΔ63 ura3Δ0 KanMX:GAL-3HA-SMC5 bar1::URAca 6His-Flag-SMT3:hphNT MMS21-3HA-UBC9:HIS3* |
| YTR3135 | *MATa bar1Δleu2-3,112 ura3-52 his3-Δ200 trp1-Δ63 ade2-1 lys2-801 pep4 his3::pHIS3-lacI-NLS-GFP:HIS3 CEN3:256xlacO:URA3 mms21Δc:natNT* |
|  |  |
| **Plasmids** |  |
| pTR1094 | *YCplac22-ADH1p-SMC5:9myc* |
| pTR1621 | *YCplac22-ADH1p-SMC5(K75I):9myc* |
| pNC1828 | *YCplac22-ADH1p-SMC5(D1014A):9myc* |
| pTR1967 | *YCplac22-ADH1p-SMC5(P271E)-9myc* |
| pTR1969 | *YCplac22-ADH1p-SMC5(P305E)-9myc* |
| pNC2089 | *pET28a-6His-T7-SMC5(K75I)* |
| pNC2094 | *pET15b-6his-HA-MMS21(M1,M2)* |
| pTR2158 | *YCplac22-ADH1p-SMC5(P393DLEL)-9myc* |
| pNC2279 | *pET15b-6his-HA-MMS21* |
| pPM2750 | *YCplac22-ADH1p-SMC5(S3)-9myc* |
| pCG2788 | *YCplac22-ADH1p-SMC5(S1)-9myc* |
| pCG2821 | *YCplac22-ADH1p-SMC5(S2)-9myc* |
| pTR3154 | *pRS315-NSE4-9myc* |
| 28S1 | *pET28a-6His-T7-SMC5* |
|  |  |
